# Nitrogen‐Functionalized Lignin: Current Status, Applications, and Challenges

**DOI:** 10.1002/cssc.202500607

**Published:** 2025-07-09

**Authors:** Jiansong Chen, Kun Liu, Haishun Du, Xuejun Pan

**Affiliations:** ^1^ Department of Biological Systems Engineering University of Wisconsin‐Madison 460 Henry Mall Madison WI 53706 USA

**Keywords:** chemical modifications, composites, lignin functionalizations, lignin valorizations, nitrogen‐functionalizations

## Abstract

Lignin—a natural, abundant, and renewable biopolymer—has garnered increasing attention for its intrinsic sustainability and versatile chemical architecture. The abundance of reactive functional groups within lignin enables its modification to meet specific application requirements. Nitrogen‐functionalized lignin (N‐lignin), in particular, has emerged as a focal point in contemporary research, providing a pathway to introduce nitrogen functionality through chemical modifications, grafting copolymerization, or physical blending with nitrogen‐containing materials. These strategies significantly enhance the performance of the lignin, making it suitable for diverse applications such as catalysts, antifouling agents, nitrogen‐enriched fertilizers, and antibacterial materials. This review offers a comprehensive examination of current methodologies for the nitrogen functionalization of lignin. It also delves into the applications of these functionalized materials in diverse fields, including environmental remediation, biomedicine, energy, catalysis, and agriculture. The analysis highlights the potential of N‐lignin to drive sustainable innovations while addressing practical challenges, such as scalability, structural heterogeneity, and process optimization. By synthesizing recent advancements and identifying ongoing challenges, this review provides a roadmap for lignin valorization, emphasizing its transformative role in fostering sustainable technologies and materials.

## Graphical abstract

This Table of Contents graphic illustrates strategies for nitrogen‐functionalizing lignin via physical blending, chemical modification, and grafting copolymerization, enabling advanced applications in energy, environment, and biomedicine. The central concept highlights the synergy between lignin valorization and nitrogen chemistry for functional material design.

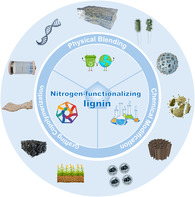

## 1 Introduction

**Figure 1:**
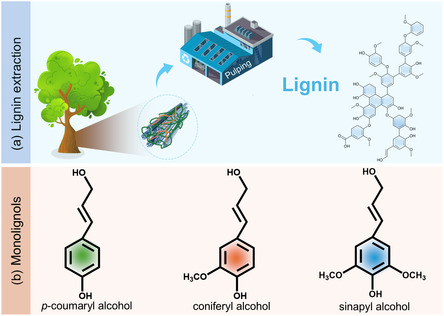
a) Illustration of lignin extraction and b) three monolignols.

Lignin is the most abundant aromatic biopolymer on Earth and is critical in providing rigidity and strength to plant cell walls.^[^ ^1^ ^]^ Comprising phenylpropane units interconnected in a complex three‐dimensional network, lignin accounts for 15%–35% of the dry weight of wood and 14%–24% in herbaceous plants.^[^ ^2^ ^]^ Its biodegradability, renewability, and environmental friendliness have garnered increasing interest in sustainable material development.^[^ ^3^ ^]^ Despite its vast potential, only a fraction of the 26 million tons of lignin extracted annually by the pulp and paper industry is utilized for material and chemical applications, with over 98% incinerated for energy recovery (**Figure** **1**a).^[^ ^4^ ^]^ This not only wastes biomass resources but also contributes to greenhouse gas emissions.^[^ ^5^ ^]^

Lignin can be classified based on its extraction methods, such as kraft, sulfite, soda, and organic solvent lignin.^[^ ^6^ ^]^ Lignin is primarily derived from three monolignols, including *p*‐coumaryl alcohol, coniferyl alcohol, and sinapyl alcohol, which correspond to the corresponding aromatic units: *p*‐hydroxyphenyl (H), guaiacyl (G), and syringyl (S), respectively (Figure 1b).^[^ ^7^ ^]^ The proportions of these units vary depending on plant species. Lignin structure is abundant in functional groups, including carbonyl, phenolic hydroxyl, and alcoholic hydroxyl groups.^[^ ^8^ ^]^ These intrinsic characteristics present great potential for developing lignin‐based functional materials.^[^ ^9^ ^]^

Among the strategies to enhance the versatility of lignin, nitrogen functionalization has emerged as a particularly promising approach.^[^ ^10^ ^]^ The elemental composition of lignin varies with the extraction methods but primarily consists of carbon, hydrogen, and oxygen, with only trace amounts of nitrogen and sulfur.^[^ ^11^ ^]^ Given the naturally low nitrogen content in lignin,^[^ ^12^ ^]^ incorporating nitrogen‐containing functional groups, such as amines, amides, and nitro groups, or integrating nitrogen‐rich materials with lignin, can significantly modify its physicochemical properties and expand its applications across various fields, including agriculture,^[^ ^13^ ^]^ biomedical,^[^ ^14^ ^]^ catalysis,^[^ ^15^ ^]^ energy,^[^ ^16^ ^]^ and environmental remediation.^[^ ^17^ ^]^

Nitrogen functionalization of lignin can be achieved through three primary approaches: physical blending,^[^ ^18^ ^]^ grafting copolymerization,^[^ ^19^ ^]^ and chemical modification.^[^ ^20^ ^]^ Physical blending involves noncovalent interactions such as hydrogen bonding, π–π stacking, and electrostatic interactions between lignin and nitrogen‐containing materials.^[^ ^21^ ^]^ These nitrogen‐containing materials include small molecules, synthetic polymers, natural polymers, and metal‐organic frameworks.^[^ ^22^ ^]^ This strategy enhances properties such as biodegradability, antimicrobial properties, electrical sensing capabilities, and water retention. Prominent examples include the incorporation of chitosan,^[^ ^23^ ^]^ chitin,^[^ ^24^ ^]^ polyimide,^[^ ^25^ ^]^ and polypyrrole^[^ ^26^ ^]^ matrices, making it particularly suitable for biomedical, electrochemical, and sustainable material applications.

Grafting copolymerization involves the covalent attachment of nitrogen‐rich compounds to lignin, imparting specialized functionalities.^[^ ^27^ ^]^ Lignin contains reactive functional groups, including phenolic hydroxyl, aliphatic hydroxyl, and carboxyl groups, which enable grafting through various polymerization mechanisms.^[^ ^28^ ^]^ Free‐radical polymerization, commonly initiated by benzoyl peroxide or ammonium persulfate, facilitates the reaction of phenolic groups of lignin with nitrogen‐containing monomers such as acrylamide and polyaniline, yielding stable hybrid materials.^[^ ^29^ ^]^ Alternatively, polymerization process based on a condensation reaction enables hydroxyl or carboxyl groups of lignin to react with nitrogen‐rich molecules such as urea, melamine, or polyimides, forming ether, amide, or urethane linkages that improve thermal stability, mechanical strength, and hydrophilicity.^[^ ^30^ ^]^

In contrast, chemical modification techniques offer a more direct and tunable approach by introducing covalent nitrogen‐containing functional groups onto backbone of lignin.^[^ ^31^ ^]^ This process introduces nitrogen‐containing functional groups into lignin via chemical reactions. For example, the Mannich reaction introduces amino groups by reacting lignin with formaldehyde and a secondary amine, improving its solubility, reactivity, and chelation potential.^[^ ^32^ ^]^ Similarly, the Williamson etherification reaction forms nitrogen‐containing esters through interactions with alkyl halides and bases, expanding applications of lignin in bio‐based materials, catalysis, and functional coatings.^[^ ^33^ ^]^

Based on the nitrogen functionalization of the lignin, significant advancements have been made in exploring its potential for novel applications and enhancing the performance of existing ones. This review offers a comprehensive overview of N‐lignin, highlighting recent nitrogen‐unctionalization techniques, its diverse applications across environmental, biomedical, and engineering sectors, and the associated challenges in its utilization. By addressing these aspects, this article aims to advance the field of lignin valorization and foster the development of sustainable materials and technologies.

## 2 Methods of Preparing N‐Lignin

### 2.1 Physical Blending

Physical blending of lignin with nitrogen‐containing materials presents a straightforward strategy to enhance the functionalities of lignin and expand its applications. By combining lignin with compounds such as chitosan, chitin, gelatin, or polypyrrole, the resulting composites synergistically integrate lignin with nitrogen‐based functionalities. This integration enhances mechanical strength, improves adsorption capacity, and supports diverse applications, including water treatment,^[^ ^34^ ^]^ adhesives,^[^ ^35^ ^]^ biomedical uses,^[^ ^36^ ^]^ and catalytic processes.^[^ ^37^ ^]^ Furthermore, this approach eliminates the need for complex chemical reactions, ensuring scalability and economic feasibility.

**Figure 2:**
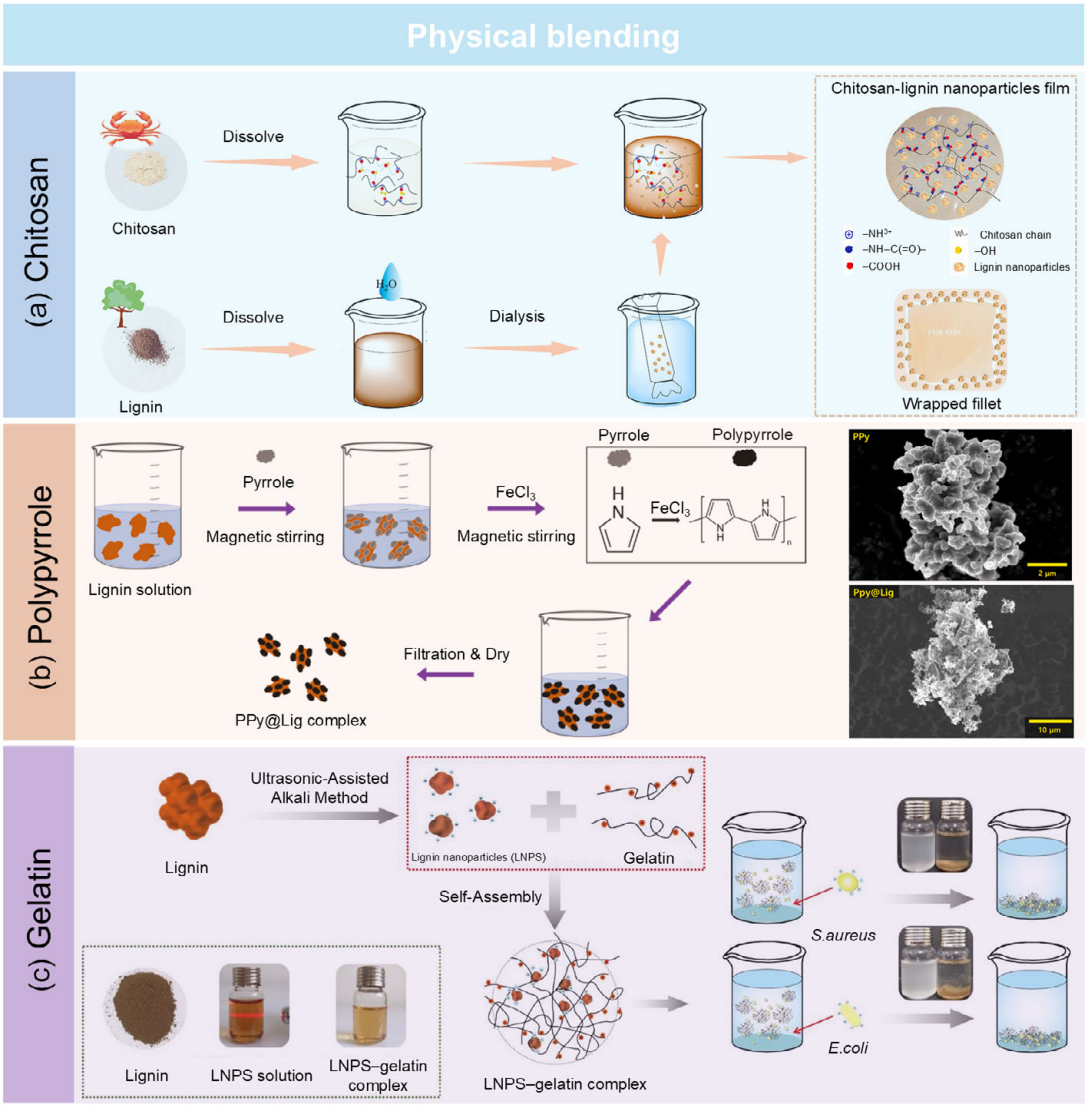
Nitrogen functionalization of lignin by physical blending with a) chitosan. Adapted with permission.^[^ ^40^ ^]^ Copyright 2023, Elsevier. b) Polypyrrole. Adapted with permission.^[^ ^26^ ^]^ Copyright 2022, Elsevier. c) Gelatin. Adapted with permission.^[^ ^41^ ^]^ Copyright 2018, Elsevier.

Natural nitrogen‐containing biopolymers, such as chitosan and gelatin, have been widely explored for blending with lignin to develop multifunctional composite materials. Chitosan is a polysaccharide rich in amine and hydroxyl groups, and it is derived by the deacetylation of chitin. This versatile biopolymer is widely utilized in fields like biomedicine, agriculture, and food packaging.^[^ ^38^ ^]^ Due to its unique properties, researchers have developed lignin‐chitosan composites for a variety of applications.^[^ ^39^ ^]^ For example, Zhang et al^[^ ^40^ ^]^ embedded lignin nanoparticles (LNPs) into a chitosan matrix to create a composite film, which demonstrated enhanced water resistance and mechanical strength. Despite chitosan being the continuous phase, the synergistic interaction with lignin significantly contributed to the UV‐blocking, antibacterial, and antioxidant capabilities of the composite film (**Figure** **2**a). This highlights how nitrogen‐rich matrices can complement the phenolic structure of lignin, extending its applicability in food packaging and preservation.

In addition to chitosan, gelatin has also been explored for blending with lignin due to its excellent biocompatibility and film‐forming properties. Yin et al.^[^ ^41^ ^]^ developed a lignin‐gelatin complex as a novel flocculant for bacterial removal. Using the lignin extracted from switchgrass using an ultrasonic‐assisted alkali method, the researchers synthesized LNPs and combined them with gelatin to improve flocculation efficiency. The resulting LNPs‐gelatin complex demonstrated an impressive ability to capture both Gram‐positive (*Staphylococcus aureus*) and Gram‐negative (*Escherichia coli*) bacteria, achieving a flocculation efficiency exceeding 95% within 30 min at pH 4.5. This study highlighted the synergistic effect of the cationic nature of gelatin and the structural stability of lignin, which facilitated bacterial aggregation and sedimentation. With its high efficiency, biodegradability, and scalability, this lignin‐gelatin composite offers a promising eco‐friendly solution for wastewater treatment and microbial removal. The blending of lignin with nitrogen‐containing biopolymers represents not only a sustainable material design strategy but also a promising platform to fine‐tune interfacial interactions and functional performance.

Apart from biopolymers, synthetic polymers such as polypyrrole (PPy) has been blended with lignin to fabricate conductive lignin‐based composites. PPy is a widely studied intrinsically conductive polymer with a conjugated π‐electron system, offering excellent electrical conductivity, environmental stability, and biocompatibility.^[^ ^42^ ^]^ These properties make PPy‐lignin composites promising for applications in energy storage, sensors, biomedical devices, and electromagnetic shielding. Hur et al.^[^ ^26^ ^]^ developed a one‐pot chemical oxidative polymerization process in which pyrrole was polymerized in the presence of lignin platelets to form PPy@lignin composites (Figure 2b). The polymerization primarily occurred on the surface of lignin through physical deposition driven by polar interactions. The resulting composite exhibited remarkable ammonia‐sensing capability, displaying a linear response to NH_3_ at concentrations as low as 10–100 nM. Additionally, the PPy@lignin composite demonstrated excellent adsorption capacity for heavy metal ions, particularly ^133^Cs, with a rapid 99% removal efficiency within 2 h. The adsorption behavior followed pseudo‐second‐order kinetics and the Freundlich isotherm model, suggesting a heterogeneous surface interaction. These findings highlight the synergistic effect of lignin and polypyrrole, where lignin provides structural stability and functional groups for adsorption, while PPy ensures electrical responsiveness, paving the way for sustainable, multifunctional materials in environmental remediation.

Moreover, advanced hybrid systems incorporating nitrogen‐containing metal‐organic frameworks such as ZIF‐8 have further expanded the scope of physical blending approaches. ZIF‐8 is a composite of zinc ions (Zn^2+^) and 2‐methylimidazolate ligands. It exhibits a zeolite‐like structure with high thermal and chemical stability, as well as high porosity, making it widely used in gas separation, adsorption, catalysis, and drug delivery.^[^ ^43^ ^]^ Xu et al.^[^ ^44^ ^]^ prepared a hybrid nanocomposite of ZIF‐8 and lignin via a one‐step, room‐temperature method. Specifically, 2‐methylimidazole and zinc nitrate were mixed in methanol, with varying amounts of lignin added as a templating and functionalizing agent. The reaction mixture was stirred, ultrasonicated, and then aged without stirring for 24 h. This approach preserved the crystalline integrity of the ZIF‐8 framework while incorporating lignin, resulting in a composite with enhanced adsorption capacity for methyl violet, reaching ≈1000 mg g^−1^. Despite its impressive adsorption performance, the relatively high cost of ZIF precursors and the lack of recycling studies may restrict its broader environmental application.

Overall, physical blending offers a facile and scalable route for integrating nitrogen‐containing functionalities into lignin‐based materials, with significant improvements in mechanical strength, antibacterial activity, electrical conductivity, and adsorption capacity. However, most current studies focus on demonstrating feasibility rather than elucidating the structure‐property relationships or interface dynamics that govern composite performance. From our perspective, further investigations into the molecular‐level interactions—such as hydrogen bonding, π–π stacking, and electrostatic forces—between lignin and nitrogen‐containing components are critical for advancing rational composite design.

### 2.2 Grafting Copolymerization

While physical blending provides a simple and scalable route, it often relies on weak interactions. To achieve stronger interfacial bonding and more tailored functionalities, grafting copolymerization offers a promising alternative. Grafting copolymerization is a technique that involves covalently attaching polymer chains onto a backbone polymer.^[^ ^45^ ^]^ Various nitrogen‐rich compounds, such as polyaniline, polyethyleneimine, and urea, have been explored for their potential to react with lignin and introduce new functionalities, such as improved mechanical properties, fluorescence, and biological activity.

**Figure 3:**
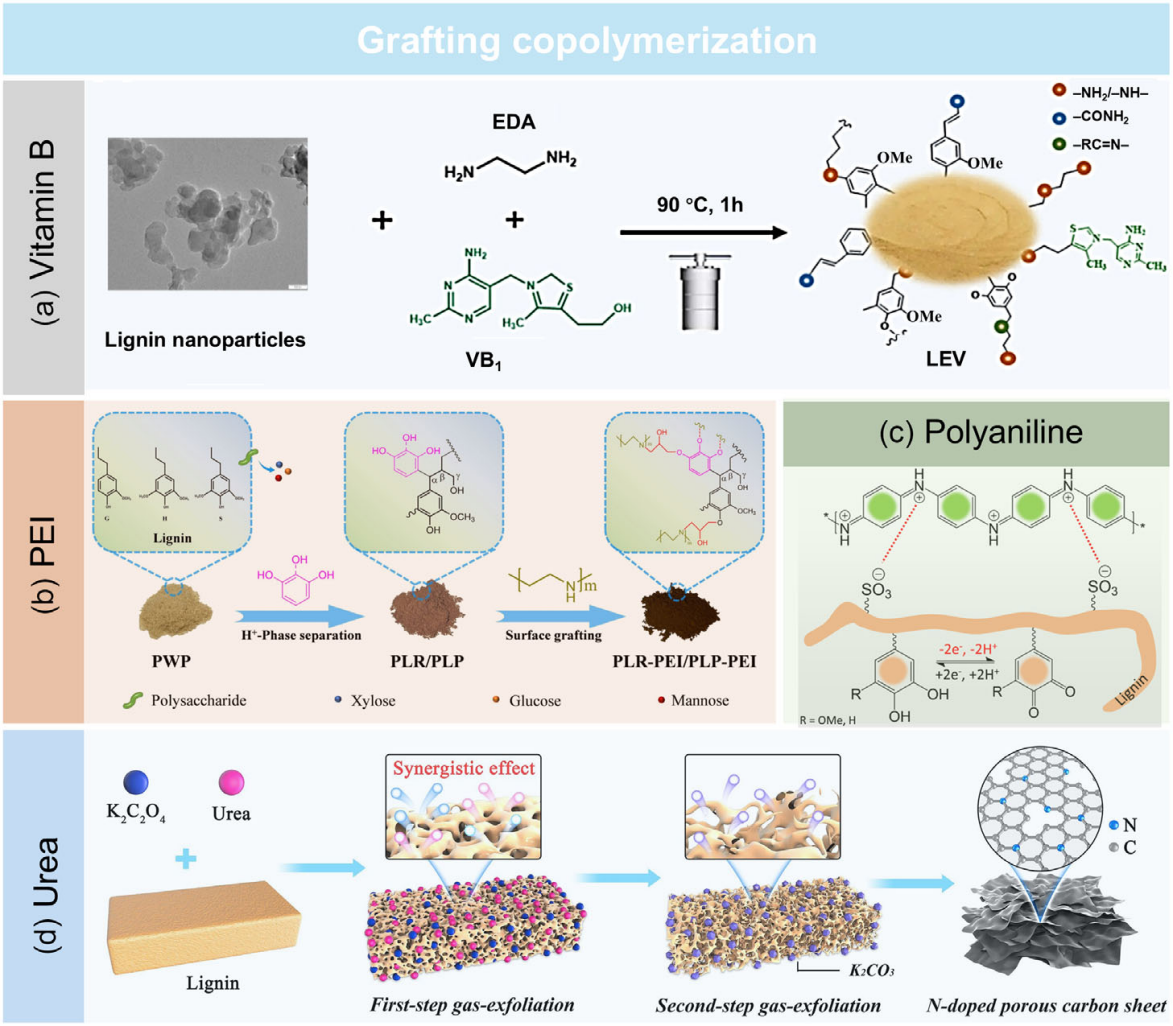
Nitrogen functionalization of lignin crosslinking with a) vitamin B. Adapted with permission.^[^ ^46^ ^]^ Copyright 2024, American Chemical Society. b) PEI. Adapted with permission.^[^ ^47^ ^]^ Copyright 2024, Elsevier. c) Polyaniline. Adapted with permission.^[^ ^29^ ^]^ Copyright 2021, American Chemical Society. d) Urea. Adapted with permission.^[^ ^48^ ^]^ Copyright 2024, Elsevier.

For instance, Yang et al.^[^ ^46^ ^]^ synthesized lignin‐based nanohybrids by grafting vitamin B1 (VB1) onto LNPs through a hydrothermal process, forming LNPs grafted with vitamin B1 hybrid nanoparticles (LEV)  with significantly enhanced fluorescence and biological activity (**Figure** **3**a). The addition of VB1 and ethylenediamine introduced imine and amide groups, increasing the positive charge density and promoting electrostatic interactions with bacterial membranes. These modifications achieved a remarkable 99.9% antibacterial efficiency and a 97.8% free radical scavenging rate. Furthermore, incorporating LEV into polyvinyl alcohol hydrogels through a freeze‐thaw method significantly improved their mechanical properties. The compression modulus increased from 51.2 to 182.1 kPa, and the tensile strength rose from 90 to 140 kPa, while bioactivity was also improved. These results underscore the potential of N‐lignin for applications in biomedical materials, smart coatings, and multifunctional hydrogels.

Besides small bioactive molecules, grafting of polymeric amines such as polyethyleneimine has been employed to further enhance the reactivity and adsorption capacity of lignin. Ding et al.^[^ ^47^ ^]^ demonstrated that the chemical crosslinking of lignin with nitrogen‐rich polyethyleneimine significantly enhanced its adsorption capacity and stability for heavy metal removal. They first used an acid hydrolysis‐phase separation method to modify poplar lignin with pyrogallol, thereby introducing abundant phenolic hydroxyl groups. These phenolic groups served as reactive sites for subsequent functionalization. In the next step, polyethyleneimine was grafted onto the phenol‐enriched lignin via atom transfer radical polymerization and crosslinking using epichlorohydrin, which covalently linked the amino‐rich polyethyleneimine chains to the lignin backbone. As a result, the aminated lignin polyphenol contained both phenolic hydroxyl and amine groups. These functional groups enabled strong electrostatic interactions, complexation, and redox activity toward Cr(VI), facilitating its reduction to Cr(III). This synergistic functionalization led to an exceptional adsorption capacity of 598.8 mg g^−1^, along with excellent regeneration properties over multiple cycles (Figure 3b).

In parallel, electrically conductive polymers such as polyaniline (PANI) have also been covalently linked to lignin to enable energy storage and sensing applications. The incorporation of PANI into lignin represents a promising strategy for enhancing electrochemical properties, as demonstrated by Dianat et al.^[^ ^29^ ^]^ in their study on lignosulfonate‐based supercapacitors. By leveraging the redox activity of lignin and the conductive nature of PANI, this hybrid material achieved high specific capacitance and excellent cycling stability. The structural synergy between lignosulfonate and polyaniline enhances ion transport, leading to a specific capacitance of 1200 F g^−1^ at 1 A g^−1^, surpassing many existing biopolymer‐based energy storage materials. Additionally, the introduction of sulfonated lignin not only provides structural stability but also acts as an active dopant, improving charge storage through proton‐assisted redox reactions. These findings underscore the potential of chemically crosslinked lignin‐polyaniline composites for sustainable energy storage applications, paving the way for the development of high‐performance, environmentally friendly supercapacitors. (Figure 3c). Complementary to conjugated polymers, nitrogen‐rich small molecules like urea have been adopted to improve porosity and electrochemical performance in lignin‐derived carbon materials. Xue et al.^[^ ^48^ ^]^ presented a compelling example of how nitrogen‐rich compounds, particularly urea, can significantly enhance the functional properties of lignin‐derived materials. They introduced a novel urea‐boosted gas‐exfoliation method to synthesize lignin‐derived porous carbon, ideal for use in zinc ion hybrid supercapacitors (Figure 3d). By incorporating urea into the activation process alongside potassium oxalate (K_2_C_2_O_4_), they achieved a remarkable increase in surface area, from 592 to 1949 m^2^ g^−1^, while successfully converting thick carbon bulks into layered carbon sheets. This structural transformation, coupled with N and O codoping, significantly improved the adsorption energy of zinc ions, optimizing ion transport and electrochemical performance. The resulting material exhibited an impressive energy density of 126.7 Wh kg^−1^ at a power density of 79.4 W kg^−1^, positioning it as a promising candidate for next‐generation energy storage devices.

In addition to its role in energy storage, lignin's functional versatility extends to the reinforcement of hydrogel‐based soft materials. Chen et al.^[^ ^30^ ^]^ developed lignin‐based nanocomposite hydrogels via in situ polymerization, using LNPs as crosslinking junctions within a polyacrylamide matrix. The incorporation of lignin not only enhanced the tensile strength from 38 to 110 kPa and elongation at break from 190% to 750% but also developed a robust hydrogen‐bonded network that enabled exceptional mechanical resilience and recoverability. This improvement is attributed to the phenolic hydroxyl groups in lignin, which facilitate both covalent and noncovalent interactions with polymer chains, thereby reinforcing the hydrogel structure. The study further revealed that increasing lignin content (up to 23.5%) significantly boosted fracture stress from 0.04 to 7.87 MPa, highlighting the reinforcing role of lignin in composite hydrogels. These chemically crosslinked lignin‐polymer networks provide enhanced mechanical durability and energy dissipation capabilities, paving the way for applications in biomedical materials, soft robotics, and tissue engineering scaffolds.

Collectively, these studies underscore the transformative potential of N‐lignin materials across a broad spectrum of applications. Despite these promising advancements, several key challenges remain. Most recent studies focus on functional performance improvements but provide limited insight into the underlying reaction mechanisms, grafting efficiencies, and structure‐function relationships of the resulting lignin‐based copolymers. Establishing a clear correlation between the degree of nitrogen functionalization, the resulting interfacial interactions, and the targeted performance metrics will be essential for rational material design. Moreover, many grafting systems rely on toxic crosslinkers or harsh reaction conditions, which may limit scalability and sustainability. Future efforts should aim to develop greener, more controllable copolymerization strategies to precisely tailor functionality while preserving lignin's inherent biocompatibility and environmental advantages.

### 2.3 Chemical Modification

In addition to employing physical blending and grafting copolymerization with nitrogen‐containing materials, chemical modification has become a widely adopted and effective strategy for enhancing the functionalities of lignin.

There are various nitrogen‐containing functional groups, such as amines, imines, amides, nitro groups, isocyanates, imidazoles, and more, and each possesses unique electronic properties, reactivity, and applications, making them valuable for specific purposes. For instance, primary amines (–NH_2_) have demonstrated a strong affinity for adsorption processes due to their ability to form hydrogen bonds and interact with negatively charged species, making them ideal for use in water purification, heavy metal ion removal, and dye adsorption.^[^ ^49^ ^]^ In contrast, quaternary amines (NR_4_^+^) are well‐known for their antibacterial properties, which are highly advantageous in biomedical applications, such as antifouling coatings, disinfectants, and drug delivery systems.^[^ ^50^ ^]^ Moreover, imidazole‐based functionalization has attracted significant interest in catalysis and electrochemical applications, as imidazole rings can enhance electron transfer and stability, making them valuable in energy storage devices, such as supercapacitors and batteries.^[^ ^51^ ^]^ Similarly, amide (–CONH_2_) and isocyanate (–NCO) groups enhance the mechanical strength and thermal stability of lignin‐based materials, contributing to their use in composites, adhesives, and coatings.^[^ ^52^ ^]^ Below, we will introduce each of these chemical modification strategies, discussing their principles, reagents involved, structural transformations, and potential applications.

**Figure 4:**
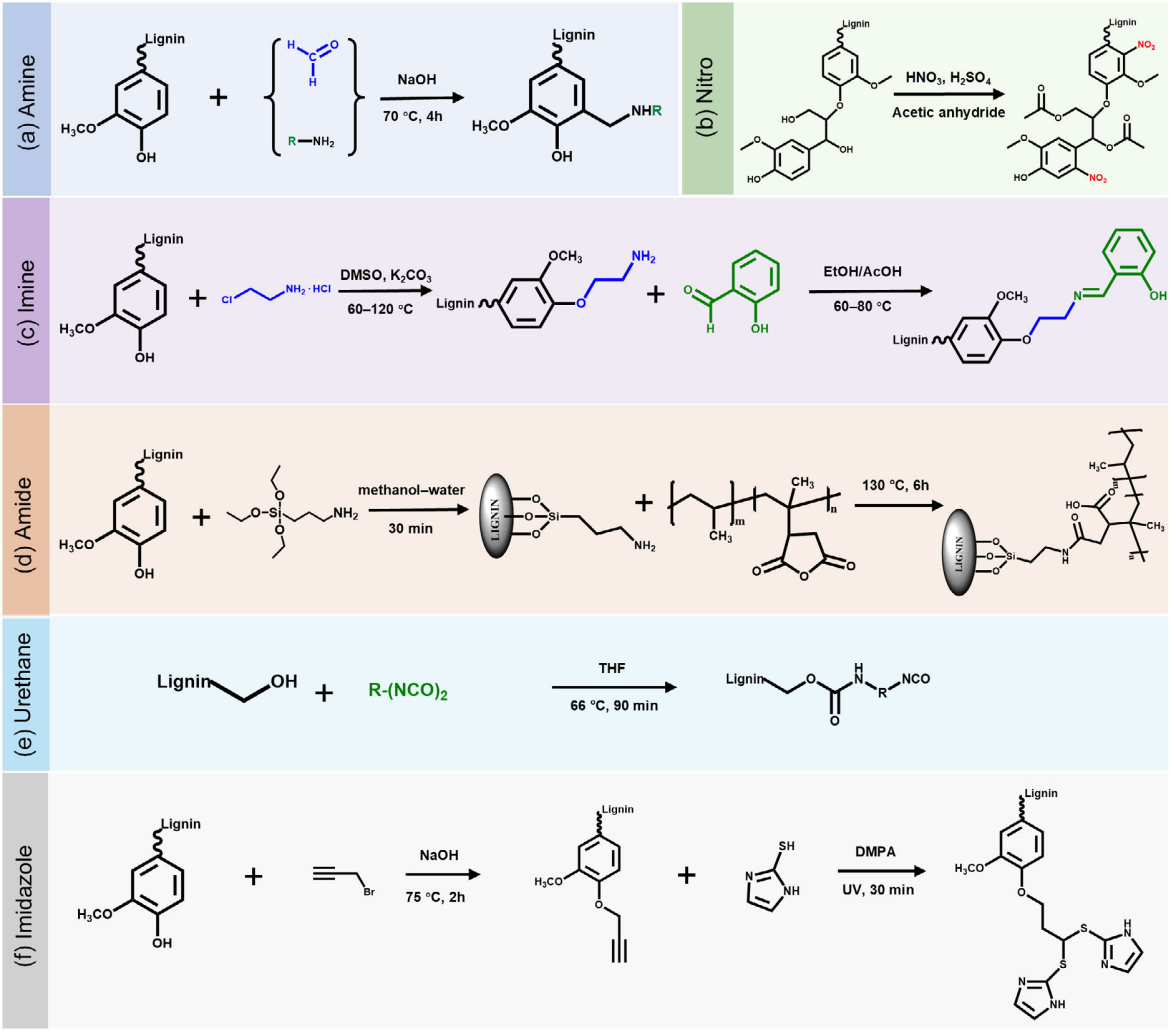
Chemical modification of lignin with nitrogen‐functional groups.

*Amines.* Amination can introduce primary amines (–NH_2_), secondary amines (–NH–), tertiary amines (–N=) or quaternary ammonium groups (NR_4_^+^) into lignin. This modification enhances the reactivity, hydrophilicity, and adsorption capacity of lignin. Among the various amination methods, the Mannich reaction is the most widely used. It introduces an amine functional group on the lignin molecules. Specifically, the reaction involves the free ortho positions of lignin phenolic moieties, formaldehyde, and an amine (**Figure** **4**a). However, this method is limited by its grafting ratio due to the availability of active sites. Additionally, the use of formaldehyde raises concerns about the safety and sustainability of this method.

To overcome these limitations, etherification‐based approaches have gained attention due to their ability to target a broader range of reactive sites. For instance, Chen et al.^[^ ^53^ ^]^ reported an amination strategy for kraft lignin using 2‐chloroethylamine hydrochloride (CEH) in an aqueous NaOH system via Williamson etherification. In this approach, 1 g of lignin was dissolved in 1.5 M NaOH and reacted with 6.44 g of the CEH at 80 °C for 24 h. The hydroxyl groups on lignin underwent nucleophilic substitution with CEH, resulting in the formation of ether linkages and successful incorporation of primary amine groups. The aminated lignin exhibited a nitrogen content of up to 4.37%. Compared with the Mannich reaction, this approach avoids the use of toxic formaldehyde and allows for modification at both phenolic and aliphatic hydroxyl sites, enabling greater flexibility in designing lignin‐based functional materials.

In addition, Pan et al.^[^ ^54^ ^]^ introduced a two‐step epoxidation‐amination strategy, where lignin was first epoxidized using epichlorohydrin and then reacted with propane diamine. In the first step, lignin was epoxidized using epichlorohydrin in 12 wt% NaOH aqueous solution at 50 °C for 8 h, with a molar ratio of phenolic hydroxyl groups to epichlorohydrin set at 1:1.1. The epoxidized lignin was subsequently subjected to amination by reacting with an excess amount of propane diamine (epoxy: amine molar ratio of 1:4) at 80 °C for 4 h. This procedure effectively introduced abundant primary and secondary amine functionalities. Elemental analysis revealed nitrogen contents of up to 6.95%, reflecting a high degree of functionalization. The pre‐functionalization step significantly improved the efficiency of subsequent amine grafting, although it involves more complex procedures and harsher reaction conditions.

In contrast, Liu et al.^[^ ^55^ ^]^ reported a solvent‐free amination strategy in which phenolic hydroxyl and carboxylic acid groups in technical lignin were directly functionalized with 2‐oxazolidinone, a cyclic urethane compound that served as both reagent and solvent. The reaction was carried out at 150 °C for 2–4 h in the presence of a basic catalyst (NaOH or Na_2_CO_3_), leading to nucleophilic substitution and ring‐opening of 2‐oxazolidinone, thereby introducing aminoethyl moieties into the lignin structure. Quantitative ^31^P NMR and elemental analysis revealed that the degree of substitution reached up to 76%, with a nitrogen content as high as 7.97%. This method aligns well with green chemistry principles and demonstrates excellent reactivity, though it requires elevated temperatures.

In summary, while the Mannich reaction remains a classic method, alternative routes such as etherification, epoxidation‐amination, and solvent‐free approaches offer enhanced efficiency, broader reactivity, and better sustainability. The choice of method should therefore be guided by the desired functional group density, process safety, and final application.

*Nitro.* To further diversify the chemical functionalities of lignin, nitration has emerged as an effective approach for introducing electron‐withdrawing nitro groups. Lignin nitration has emerged as a promising strategy to introduce nitrogen functionalities and enhance its reactivity for subsequent carbonization or catalytic applications. Various nitration methods have been reported, primarily based on electrophilic aromatic substitution. Common nitrating systems include nitric acid alone or combined with acetic anhydride, sulfuric acid, or organic solvents such as dioxane. These systems generate nitronium ions (NO_2_^+^), which selectively attack electron‐rich aromatic rings of lignin. For example, Graglia et al.^[^ ^10b^ ^]^ prepared nitro‐lignin through an electrophilic aromatic substitution reaction using a nitrating mixture of nitric acid and acetic anhydride. This mixture generated nitronium ions (NO_2_^+^) as the active electrophile, which selectively attacked the electron‐rich aromatic rings of lignin, introducing nitro groups primarily at meta‐positions relative to phenolic hydroxyl group (Figure 4b). In contrast, Khabarov et al.^[^ ^56^ ^]^ used a homogeneous nitration method in aqueous–dioxane media. Their findings revealed that both nitration and oxidative degradation occurred simultaneously, with the reaction introducing approximately one nitro group per 1.5–2 phenylpropane units. Interestingly, the reaction exhibited an induction period at lower nitric acid concentrations, likely due to the formation of nitrous acid intermediates that subsequently accelerated nitration. The process also led to significant structural changes, including cleavage of side chains and formation of quinone‐type oxidation products. In summary, nitration introduces strong electron‐withdrawing groups that enhance lignin's reactivity and functional integration in redox‐active materials. However, careful control of reaction conditions is crucial, as harsh nitration environments may lead to unwanted structural breakdown, compromising lignin's integrity in certain applications.

*Imines.* Beyond nitro groups, imine functionalities offer an alternative route for enhancing lignin's coordination and reactivity through dynamic covalent bonding. Modifying lignin with imines (Schiff bases) has gained significant attention due to its ability to enhance reactivity, coordination ability, and functional versatility. These modifications make lignin valuable for various applications, particularly in adsorption, catalysis, biomedicine, and coatings. Imines are a class of compounds that contain a C=N. Lignin modification via imine chemistry typically involves the reaction between aldehyde groups and primary amines, forming Schiff base linkages. For example, Liu et al.^[^ ^57^ ^]^ reported lignin‐based vitrimers prepared using imine chemistry. First, aldehyde‐modified lignin was prepared by treating OH‐functionalized lignin with a dialdehyde via an acetalization reaction. The modified lignin then reacted with a bio‐based dimer diamine (Priamine 1075). Due to the unique properties of imine groups, the lignin‐based imine vitrimers were used as repairable, self‐cleaning, removable, and degradable coatings. In a contrasting strategy, Xia and colleagues^[^ ^58^ ^]^ first aminated the lignin via Williamson etherification using amino‐containing alkyl chlorides, and then the aminated lignin reacted with salicylaldehyde (Figure 4c). The nitrogen content of the modified lignin could reach up to 3.32%, while preserving lignin's structural integrity. These two studies illustrate the flexibility of imine chemistry: Liu's vitrimer design highlights the potential of dynamic networks for functional coatings, whereas Xia's approach emphasizes molecular‐level control through sequential functionalization. Compared to irreversible modifications, imine linkages offer reversibility and reprocessability, although their hydrolytic stability under humid or acidic conditions remains a challenge in some applications.

*Amide.* In addition to imines, amide functionalities have also been explored to improve compatibility and mechanical strength in lignin‐based composites. Amidation has emerged as an effective strategy to introduce amide functionalities into lignin, enhancing its compatibility, hydrophilicity, and reactivity. A common amidation approach employs maleic anhydride or carboxyl‐functionalized reagents to graft amide linkages onto lignin. This strategy enhances hydrogen bonding and interfacial adhesion with polymers. For instance, Yeo et al.^[^ ^59^ ^]^ reported the amidation of lignin through the reaction between amine‐modified lignin and polypropylene‐grafted maleic anhydride, resulting in enhanced interfacial adhesion with polypropylene composites (Figure 4d). This modification not only facilitated better dispersion of lignin within the polymer matrix but also significantly improved the mechanical properties of the resulting composite.

*Urethane.* Urethane, also known as carbamate (–NH–COO–), is a functional group that consists of an amide (–NH–C=O) and an ether (–O–) linkage. Urethanes are commonly formed through the reaction between an isocyanate (–N=C=O) and a hydroxyl (–OH) group, resulting in a stable urethane bond. This pathway is a cornerstone of polyurethane chemistry, widely used in creating flexible and rigid foams, coatings, and elastomers. For example, Baniasadi et al.^[^ ^60^ ^]^ synthesized a novel lignin‐based biocomposite by grafting *n*‐octadecyl isocyanate onto lignin particles, followed by copolymerization with a low‐melting‐point polyamide matrix. This process involved a urethane reaction between lignin hydroxyl groups and *n*‐octadecyl isocyanate, converting lignin into a more hydrophobic particle that could be uniformly dispersed within the polyamide matrix. The resulting biocomposite exhibited improved mechanical properties and compatibility with the matrix, positioning it as a potential substitute for petroleum‐based plastics. The hydroxyl‐rich structure of lignin enables direct reaction with diisocyanates to form urethane bonds, thereby enhancing its compatibility within polyurethane matrices. For example, Gómez‐Fernández et al.^[^ ^61^ ^]^ functionalized kraft lignin with isophorone diisocyanate (IPDI) by reacting lignin's hydroxyl groups with the secondary NCO groups of IPDI (NCO/OH = 3:1) in THF at 60 °C for 24 h, using dibutyltin dilaurate (0.1 wt%) as catalyst. The resulting product (k‐IPDI) had a grafting degree of ≈52%, as confirmed by Fourier transform infrared spectroscopy, ^13^C NMR, and elemental analysis. Flexible polyurethane foams containing 3–10 wt% of k‐IPDI were prepared via one‐shot polymerization. Compared to unmodified lignin, k‐IPDI improved foam reactivity, cell uniformity, and mechanical flexibility. Lignin extraction tests also confirmed stronger chemical bonding between k‐IPDI and the polyurethane matrix, highlighting its suitability for bio‐based PU applications. Zieglowski et al.^[^ ^62^ ^]^ demonstrated that the structure and electron affinity of the diisocyanate used for lignin functionalization significantly influence the reaction kinetics and the resulting foam morphology (Figure 4e). Their systematic study on methylene diphenyl diisocyanate (MDI), toluene diisocyanate (TDI), and hexamethylene diisocyanate (HDI)‐functionalized lignins revealed that higher reactivity (e.g., with MDI) led to improved crosslinking density and mechanical integrity in the polyurethane network. This highlights the critical role of isocyanate selection in tailoring lignin‐based prepolymers for polyurethane applications, underscoring the need for molecular‐level design in bio‐based material development.

Although urethane functionalization is more sensitive to reaction conditions and reagent selection, it offers greater structural tunability and mechanical reinforcement, particularly in polyurethane systems. The choice between these strategies depends on the desired balance between hydrophilicity, mechanical strength, and compatibility with target matrices.

*Imidazole.* Moreover, nitrogen‐containing heterocycles such as imidazole impart unique electronic and coordination properties, opening new avenues for catalysis and energy applications. Imidazole is a five‐membered heterocyclic compound with two nitrogen atoms at positions 1 and 3 in its ring structure. It is widely used in organic synthesis, catalysis, and materials science due to its unique aromaticity, nucleophilicity, and coordination properties. Unlike linear amine or amide functionalities, imidazole introduces a conjugated heterocyclic structure capable of participating in π–π stacking, hydrogen bonding, and chelation, thus expanding the functional versatility of lignin‐based materials. For instance, Qin et al.^[^ ^63^ ^]^ developed an imidazole‐modified lignin (IL) through a thiol‐alkyne click reaction to introduce nitrogen and sulfur heteroatoms into the lignin structure. The imidazole modification enabled strong chelation with cobalt salts, which facilitated the formation of an N, S codoped cobalt sulfide catalyst upon pyrolysis. This modification significantly improved the electrocatalytic activity of the material by enhancing charge‐transfer efficiency and increasing active sites for catalytic reactions. (Figure 4f). Imidazole‐functionalized lignin offers a compelling platform for next‐generation energy and catalytic applications, though challenges remain in terms of synthetic complexity and cost. Nonetheless, its potential for multifunctionality and electronic structure modulation positions it as a valuable direction for advanced lignin valorization.

## 3 Applications of N‐Lignin

**Figure 5:**
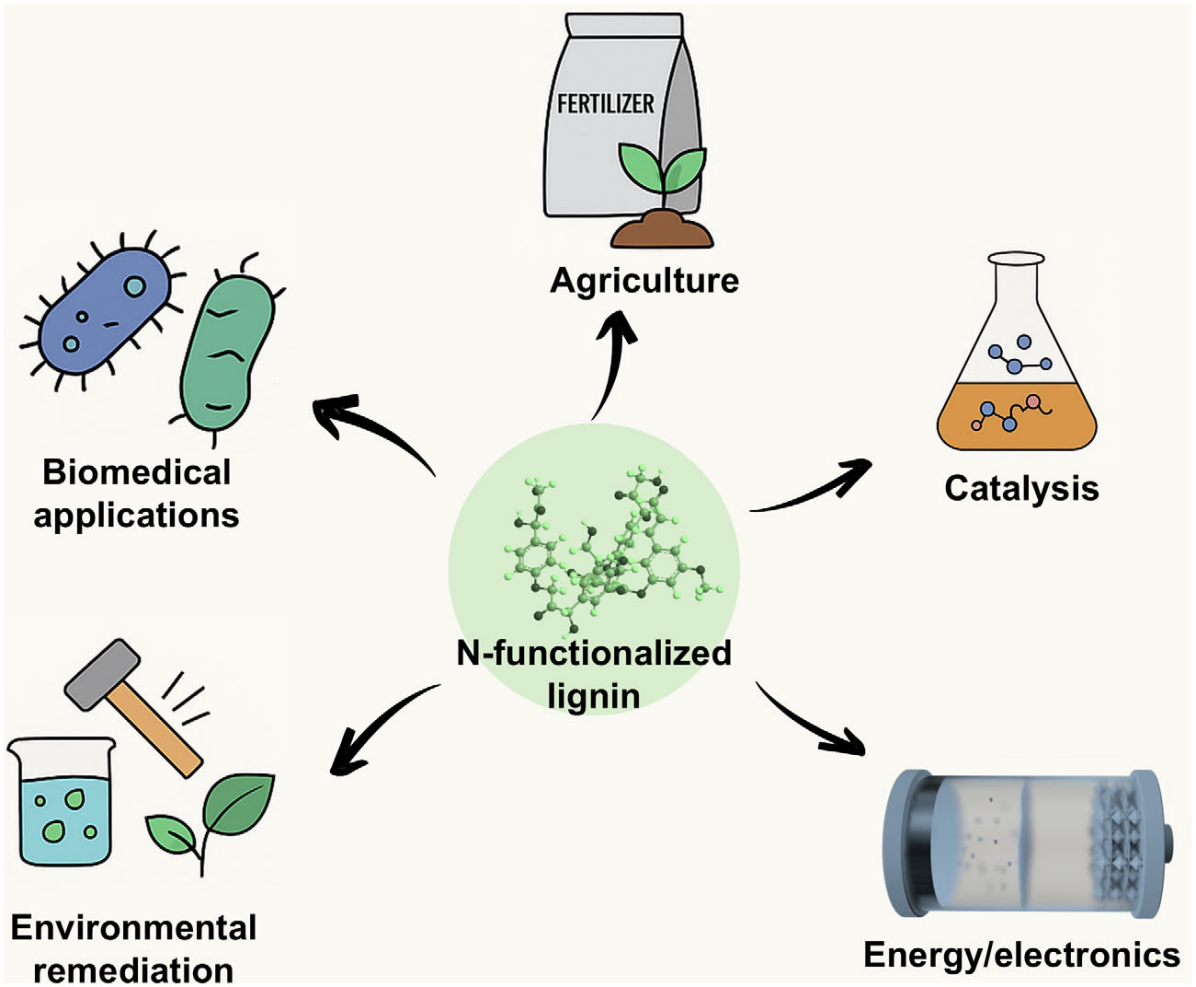
Applications of N‐lignin in biomedical applications, agriculture, catalysis, environmental remediation, and energy applications.

Owing to its enhanced physicochemical properties, N‐lignin has demonstrated considerable potential across diverse sectors, including biomedicine, agriculture, catalysis, environmental remediation, and energy. (**Figure** **5**).

In biomedical and personal care applications, N‐lignin exhibits antibacterial, antioxidant, and UV‐blocking properties, making it suitable for antimicrobial coatings, packaging materials, sunscreens, and cosmetic formulations. In the environmental sector, nitrogen functionalities enhance lignin's adsorption affinity for heavy metals and dyes, making it effective for wastewater treatment. In agriculture, its nutrient‐rich composition and slow‐release behavior support its application as a sustainable fertilizer that improves soil quality.

Beyond these, nitrogen doping improves lignin's ability to catalyze redox reactions, supporting its use as a catalyst or catalyst support in green synthesis and biomass conversion. Moreover, the improved surface activity and amphiphilic balance of N‐lignin confer valuable emulsifying properties, beneficial for applications in food processing, pharmaceuticals, and industrial formulations.

These multifunctional properties, derived from the synergy between lignin's aromatic backbone and nitrogen‐based functionality, position N‐lignin as a sustainable, high‐performance platform material with broad industrial relevance. The following sections provide a detailed overview of its specific applications in biomedical, environmental, catalytic, and energy‐related fields.

### 3.1 Antioxidant

Antioxidants play a critical role in preventing oxidative degradation in food, pharmaceuticals, polymers, and biological systems.^[^ ^64^ ^]^ There are primarily two categories of antioxidants: synthetic antioxidants and natural antioxidants.^[^ ^65^ ^]^ Synthetic antioxidants, such as propyl gallate^[^ ^66^ ^]^ and butylated hydroxyanisole,^[^ ^67^ ^]^ are widely used due to their strong antioxidant capacity, effectively preventing oxidation and prolonging the shelf life of food products, pharmaceuticals, and industrial materials. In contrast, natural antioxidants, such as carotenoids and vitamin C, are derived from plant‐based sources and play a crucial role in protecting biological systems from oxidative stress. Although synthetic antioxidants generally exhibit superior antioxidant efficiency compared to their natural counterparts, their use is under increasing scrutiny due to potential health risks. Many synthetic antioxidants have been linked to adverse effects, including toxicity and carcinogenicity, which raise serious safety concerns, especially in food and medical applications. As a result, there is an urgent demand for the identification and development of natural antioxidants that offer robust antioxidant activity while posing minimal health risks.^[^ ^68^ ^]^

Unlike synthetic antioxidants, lignin is a biocompatible, biodegradable, and natural polymer. It shows great promise as a sustainable antioxidant.^[^ ^69^ ^]^ Exploring lignin as a natural antioxidant could lead to safer and more sustainable solutions in food preservation, pharmaceuticals, and other industries where oxidative degradation is a concern.^[^ ^70^ ^]^ Lignin is rich in phenolic hydroxyl groups, allowing it to effectively scavenge free radicals and mitigate oxidative damage.^[^ ^71^ ^]^ The aromatic rings in lignin provide resonance stabilization of radicals, further enhancing its antioxidant efficiency.^[^ ^72^ ^]^ Additionally, lignin is involved in redox cycling, facilitating continuous electron transfer to counteract oxidative stress. Its ability to chelate metal ions is another vital attribute, as it inhibits the Fenton reaction responsible for generating highly reactive hydroxyl radicals. These mechanisms collectively contribute to lignin's intrinsic antioxidant activity, making it a viable candidate for use in food preservation, cosmetics, pharmaceuticals, and polymer stabilization.^[^ ^71^, ^73^ ^]^

To further improve the antioxidant capacity of lignin, various strategies have been developed, including organic solvent fractionation,^[^ ^74^ ^]^ chemical modification,^[^ ^75^ ^]^ and enzymatic treatment.^[^ ^76^ ^]^ These approaches aim to increase phenolic hydroxyl groups, and reduce the molecular weight of lignin, thereby enhancing its radical‐scavenging ability. Nitrogen functionalization stands out as a particularly promising method, involving the incorporation of nitrogen‐containing functional groups into the lignin structure. This modification can significantly improve the antioxidant activity of lignin by boosting its electron‐donating capacity and enhancing the stability of free radicals.

By introducing nitrogen‐containing functional groups, lignin gains additional active sites that participate in redox reactions, improving its efficiency in neutralizing reactive oxygen species and free radicals.^[^ ^77^ ^]^ Moreover, this functionalization can also enhance the solubility and compatibility of lignin with different formulations, making it more suitable for applications in food preservation,^[^ ^73^ ^]^ cosmetics,^[^ ^77^ ^]^ and the pharmaceutical industry.^[^ ^78^ ^]^

**Figure 6:**
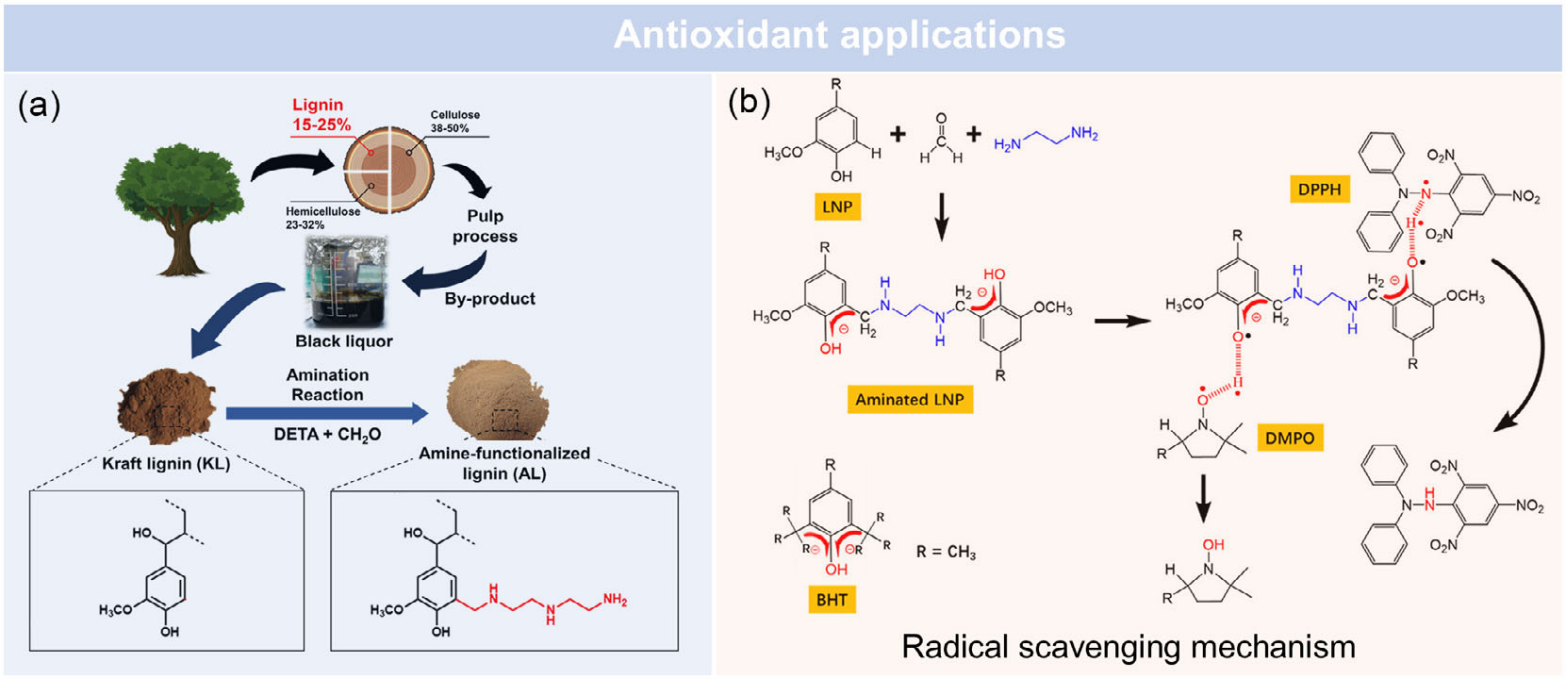
a) The application of N‐lignin in antioxidants. Adapted with permission.^[^ ^79^ ^]^ Copyright 2023, American Chemical Society. b) Radical scavenging mechanism. Adapted with permission.^[^ ^77^ ^]^ Copyright 2021, American Chemical Society.

Several studies have demonstrated the benefits of N‐lignin in both antioxidant activity and material performance. Chung et al.^[^ ^79^ ^]^ introduced primary and secondary amine groups onto lignin to enhance its radical scavenging ability and then incorporated the aminated lignin into rubber (**Figure** **6**a). This modification not only improved the antioxidant properties of lignin but also improved its compatibility with the rubber matrix, resulting in a higher crosslinking density and superior mechanical performance. Moreover, the aminated lignin actively participated in the vulcanization process, accelerating curing and minimizing ozone‐vulnerable areas within the rubber. As a result, the lignin‐incorporated rubber exhibited outstanding thermal stability, enhanced ozone resistance, and an extended fatigue life compared to conventional *N*‐(1,3‐dimethylbutyl)‐*N’*‐phenyl‐*p*‐phenylenediamine‐based rubber. These findings suggest that aminated lignin could replace synthetic antioxidants in rubber applications, offering both performance advantages and environmental benefits.

In addition to chemical modifications, increasing the surface area of lignin has also been explored as a feasible approach to enhance its antioxidant efficiency. Yang et al.^[^ ^77^ ^]^ synthesized LNPs via an acid treatment method, followed by surface amination using the Mannich reaction. The antioxidant performance of these aminated LNPs was evaluated using 1,1‐diphenyl‐2‐picrylhydrazyl and HeLa cells (a widely used immortal human cervical cancer cell line) reactive oxygen species scavenging assays. The results demonstrated that aminated LNPs exhibited significantly higher antioxidant activity compared to both unmodified LNPs and the commercial antioxidant butylated hydroxytoluene (Figure 6b). This improvement was attributed to the enhanced radical scavenging ability, enabled by the introduction of electron‐donating amine groups at the ortho‐position of the phenolic hydroxyl groups.

In summary, nitrogen functionalization significantly enhances the antioxidant performance of lignin by improving its electron‐donating capacity, redox stability, and compatibility with application matrices. These improvements make N‐lignin a compelling alternative to synthetic antioxidants, with demonstrated potential in food, cosmetic, pharmaceutical, and rubber applications.

### 3.2 Antibacterial

Bacterial contamination poses significant challenges across medical, packaging, and environmental domains, highlighting the need for safe, sustainable antimicrobial materials.^[^ ^80^ ^]^ The inherent phenolic compounds in lignin already exhibit antimicrobial characteristics, which are utilized by plants as natural biocides to defend against microbial invasions. Notably, the effectiveness of these antimicrobial properties varies depending on the functional groups attached to the phenolic fragments. For example, functional groups of phenolic fragments with oxygen (–OH, –CO, –COOH) in the side chain are less inhibitory than the isoeugenol phenolic fragment. The antimicrobial activity of isoeugenol structures has been attributed to the side chain C_α_=C_β_ double bond and the methyl group in the γ‐position.^[^ ^81^ ^]^ This isoeugenol is an isomer belonging to a group of naturally occurring phenylpropenes.^[^ ^82^ ^]^ Hence, isoeugenol structures are preferred for enhanced antimicrobial activity.

**Figure 7:**
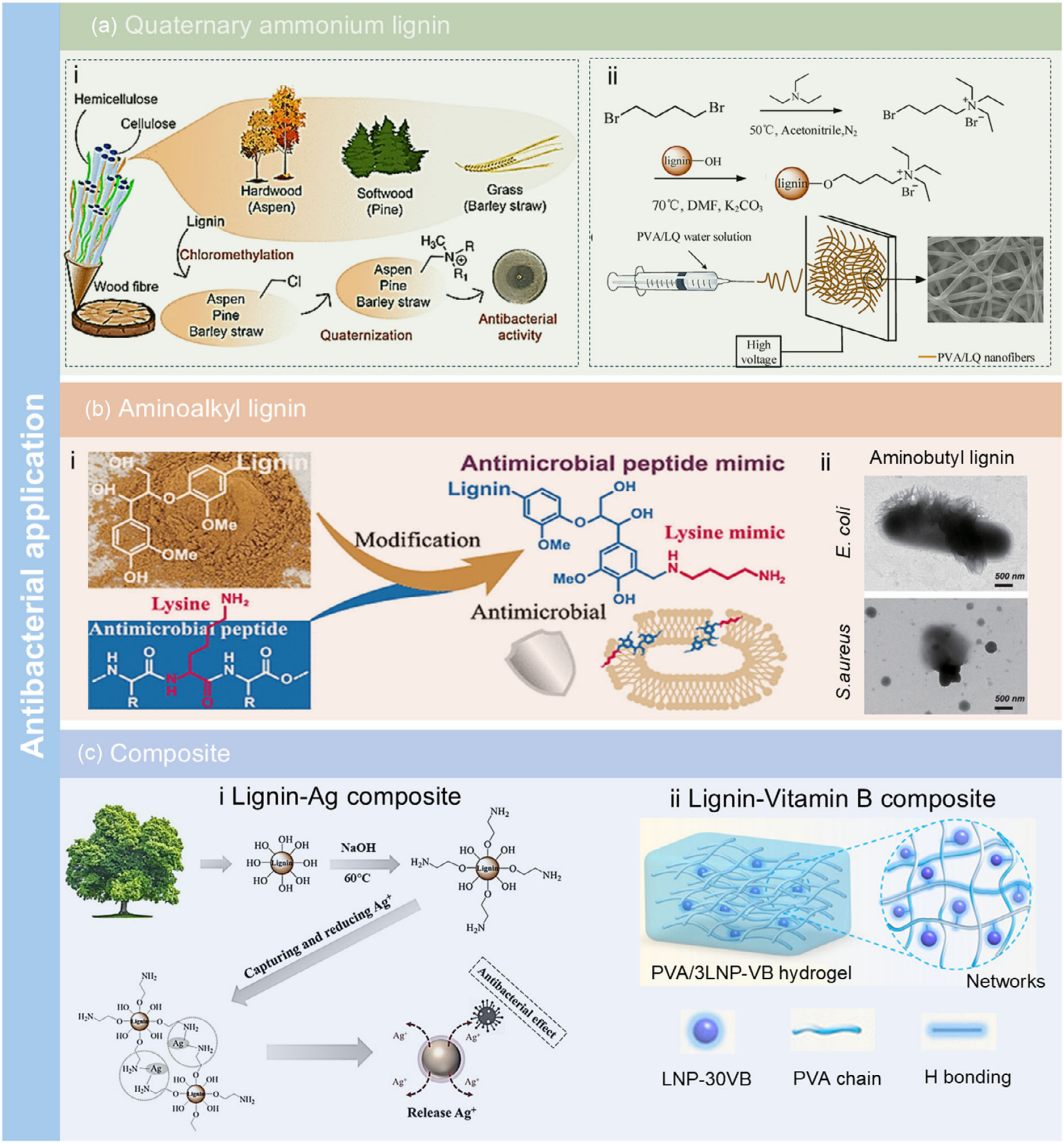
Antibacterial applications of N‐lignin by a) quaternary ammonium lignin. Adapted with permission.^[^ ^83^ ^]^ Copyright 2024 American Chemical Society under CC BY 4.0 (https://creativecommons.org/licenses/by/4.0/). Adapted with permission.^[^ ^84^ ^]^ Copyright 2023, Elsevier. b) Aminoalkyl lignin. Adapted with permission.^[^ ^85^ ^]^ Copyright 2023, American Chemical Society. c) Lignin composite. Adapted with permission.^[^ ^53^ ^]^ Copyright 2021, Elsevier. Adapted with permission.^[^ ^86^ ^]^ Copyright 2024, American Chemical Society.

While lignin itself exhibits inherent antimicrobial properties, its performance is often limited by low solubility and moderate activity. Recent research has demonstrated that nitrogen functionalization can significantly enhance lignin's antibacterial performance by introducing positive charges or coordination sites, thereby disrupting microbial membranes or interacting with bacterial enzymes. For example, quaternary ammonium compounds are widely used in antibacterial applications (**Figure** **7**ai) due to their significant antimicrobial activity, which results from the disruption of bacterial cell membranes and leakage of cellular components. This mechanism is attributed to the presence of a positively charged nitrogen atom connected with four alkyl or aryl groups, one of which is typically a lengthy hydrocarbon chain with eight or more carbon atoms, acting as a hydrophobic component. For example, Mohan et al.^[^ ^83^ ^]^ synthesized quaternary ammonium lignins derived from hardwood (aspen), softwood (pine), and grass (barley straw) using a two‐step process: chloromethylation of organosolv lignin followed by quaternization with a series of tertiary dimethyl amines (C6–C18). The chloromethylation was performed by reacting the lignin with paraformaldehyde in glacial acetic acid under HCl gas, and quaternization was carried out by heating the chlorinated lignin with alkyl dimethyl amines in acetonitrile at 80 °C for 24 h. The study demonstrated that quaternary ammonium lignins with longer alkyl chains had higher positive surface charge and improved dispersion, which correlated with enhanced antibacterial activity. Similarly, An and colleagues^[^ ^84^ ^]^ prepared a water‐soluble lignin quaternary ammonium salt by reacting kraft lignin with 6‐bromohexyl‐triethylammonium bromide in the presence of K_2_CO_3_ in DMF at 70 °C for 24 h. After dialysis and freeze‐drying, the resulting lignin exhibited good solubility (150 mg mL^−1^ in water) and a significantly increased nitrogen content of 3.35%, as confirmed by elemental analysis. The lignin quaternary ammonium salt showed excellent antibacterial performance, with a minimum inhibitory concentration of 47.90 μg mL^−1^ against *Staphylococcus aureus*, comparable to that of other macromolecular antibacterial agents (Figure 7aii).

Inspired by antimicrobial peptides, Li et al.^[^ ^85^ ^]^ mimicked lysine and synthesized aminoalkyl‐modified lignins with varying degrees of amino group substitution and hydrophobicity (Figure 7bi). The results indicated that aminobutyl lignin exhibited enhanced antimicrobial activity against *S. aureus* and *E. coli*, outperforming copper ions (Figure 7bii).

Lignin also serves as an effective platform for the complexation and stabilization of antimicrobial agents, such as silver nanoparticles (AgNPs) and vitamin B. For example, Chen et al.^[^ ^53^ ^]^ prepared aminated lignin (AL) and used it to reduce and capture Ag, thereby forming a lignin‐AgNPs hybrid with excellent antibacterial activity against gram‐positive (*Bacillus cereus*, *Staphylococcus aureus*) and gram‐negative (*Salmonella enterica*) bacteria (Figure 7ci). Meanwhile, Yang et al.^[^ ^86^ ^]^ grafted VB1 to LNPs via the Mannich reaction. VB1, known for containing an amino group and being a safe, abundant antioxidant material, enhanced the antibacterial effect of the grafted LNPs against *E. coli* and *S. aureus* (Figure 7cii). Additionally, the hydrogel made from grafted LNPs could potentially be used for wound healing, as hydrogels with good cell compatibility and blood compatibility are beneficial for wound healing and eliminating wound inflammation.

In summary, these strategies demonstrate the versatility of N‐lignin as an antimicrobial platform. Quaternary ammonium modification provides strong surface activity and broad‐spectrum antimicrobial efficacy, while aminoalkylation facilitates biomimetic membrane targeting. Metal complexation further extends lignin functionality, enabling the development of antibacterial materials. Moving forward, fine‐tuning the balance of charge density, hydrophobicity, and redox activity will be critical for developing lignin‐based antimicrobial agents tailored for biomedical and packaging applications.

### 3.3 Adsorbents

Lignin has attracted growing attention as a renewable and low‐cost adsorbent material for wastewater treatment due to its abundance and the presence of reactive functional groups such as phenolic, hydroxyl, and methoxyl moieties. These groups enable lignin to interact with and adsorb a variety of pollutants, including heavy metals, dyes, and organic compounds, making it a promising candidate for wastewater treatment applications. By harnessing the abundant and renewable nature of lignin, researchers aim to develop sustainable and cost‐effective solutions for mitigating environmental pollution and enhancing water quality in wastewater treatment processes.

**Figure 8:**
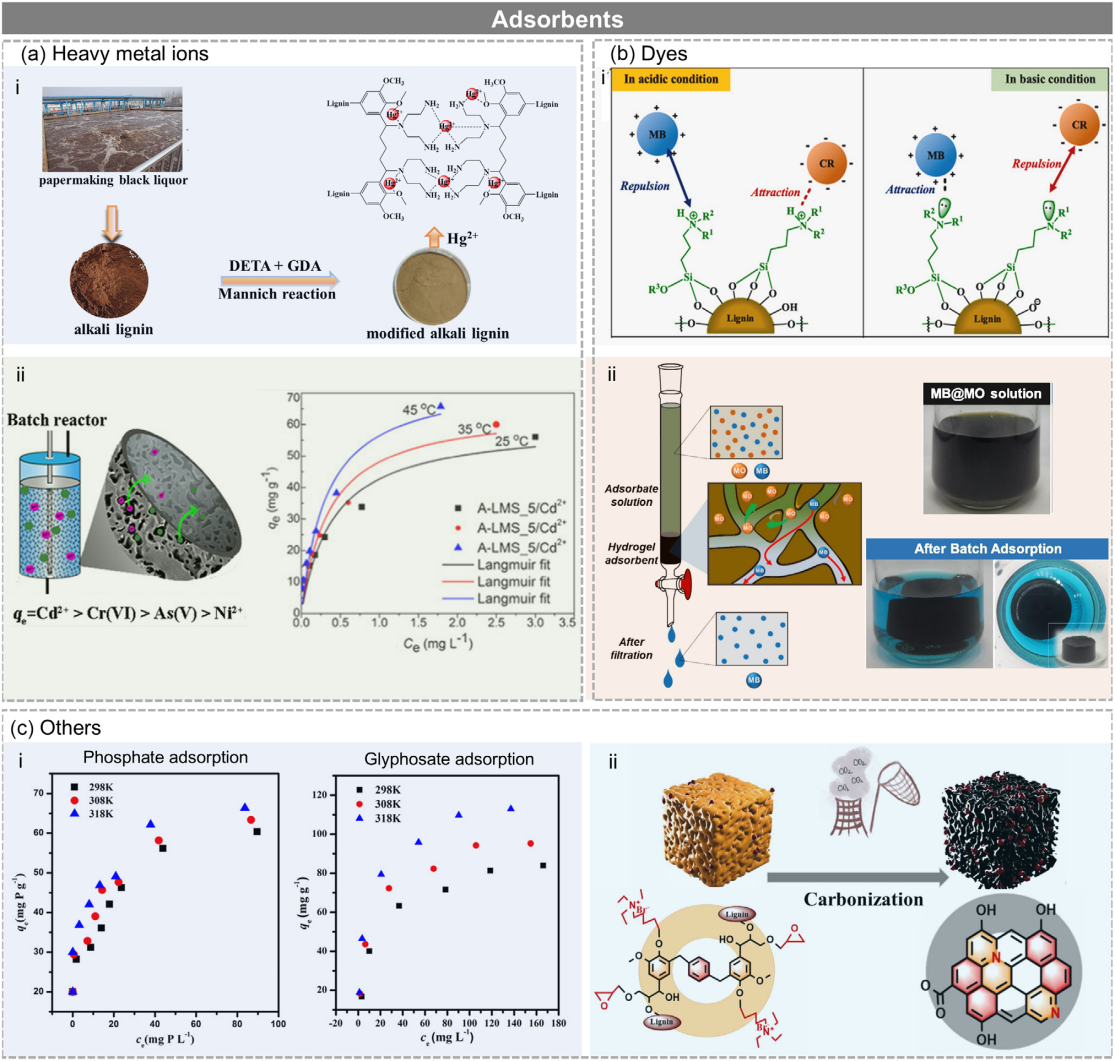
Using N‐lignin as adsorbents for a) heavy metal ions. Adapted with permission.^[^ ^88^ ^]^ Copyright 2022, Elsevier. Adapted with permission.^[^ ^89^ ^]^ Copyright 2020, Elsevier. b) Dyes. Adapted with permission.^[^ ^49b^ ^]^ Copyright 2022, Elsevier. Adapted with permission.^[^ ^90^ ^]^ Copyright 2024, Elsevier under CC BY 4.0 (https://creativecommons.org/licenses/by/4.0/). c) Others^[^ ^105^ ^]^ Adapted with permission.^[^ ^105a^ ^]^ Copyright 2021, Elsevier. Adapted with permission.^[^ ^105b^ ^]^ Copyright 2025, Elsevier under CC BY 4.0 (https://creativecommons.org/licenses/by/4.0/).

However, despite its potential, the adsorption capacity of lignin‐based adsorbents is still relatively limited compared to conventional carbonaceous materials, such as activated carbon and graphene‐based adsorbents.^[^ ^87^ ^]^ To overcome this limitation and enhance adsorption efficiency of lignin, various functionalization strategies have been explored. Among them, nitrogen functionalization has emerged as a promising approach, as nitrogen‐containing groups, particularly primary amine groups, exhibit a strong affinity toward a wide range of pollutants to create highly efficient biomass adsorbents. N‐lignin shows excellent capacity for binding toxic heavy metals through chelation or electrostatic interactions. For example, Zhou et al.^[^ ^88^ ^]^ prepared amino‐functionalized alkali lignin using dialdehyde and triamine (**Figure** **8**ai), and the N‐functionalized lignin showed exceptionally high adsorption capacity for Hg^2+^ (>500 mg g^−1^). The authors attributed this high adsorption capacity to the nitrogen‐containing functional groups grafted into the lignin, with the N atoms of amino groups sharing their lone pair of electrons with Hg^2+^ to form complexes, confirming that =N– and –NH_2_ groups on the surface of aminated alkaline lignin (AAL) acted as the main adsorption sites. Similarly, Popovic et al. prepared amino‐functionalized lignin microspheres for heavy metal ions removal. In this process, kraft lignin was copolymerized with polyethyleneimine as the grafting agent and epoxy chloropropane as the crosslinker in the presence of sodium alginate as an emulsifier. The optimized formulation using 5 wt% alginate produced highly porous microspheres (≈800 μm diameter, 68% porosity), with a high density of terminal amino groups (7.7 mmol g^−1^), significantly enhancing the adsorption performance toward Ni^2+^, Cd^2+^, As(V), and Cr(VI) ions (Figure 8aii).^[^ ^89^ ^]^

The N‐lignin also showed significant improvement in adsorption toward both cationic and anionic dyes. For example, Heo et al.^[^ ^49b^ ^]^ prepared three different types of lignin modified by silane coupling agents with different amine groups (primary, secondary, and tertiary) and compared their adsorption capacities toward both cationic and anionic dyes (Figure 8bi). The lignin modified with primary amine had the highest adsorption capacity for methylene blue and Congo red, reaching 187 and 293 mg g^−1^, respectively, followed by lignins modified with the secondary amine and tertiary amine. Additionally, the group also prepared a branched aminated lignin hydrogel using poly(ethylene glycol) diglycidyl ether as a crosslinker. Impressively, the lignin‐based hydrogel showed very high adsorption performance, especially for anionic dyes, with methyl orange adsorption reaching ≈900–930 mg g^−1^. It even maintained a high removal efficiency after 10 adsorption and desorption cycles^[^ ^90^ ^]^ (Figure 8bii).

Apart from heavy metal ions and dyes, N‐lignin has also been explored for the adsorption of other pollutants, such as 2,4,6 trinitrotoluene (TNT), phosphate, glyphosate, CO_2_, and so on.

Wastewater containing TNT has caused many serious environmental problems worldwide. Zhang et al.^[^ ^91^ ^]^ developed a novel modified lignin adsorbent for TNT, synthesizing aminated lignin through a two‐step chemical modification. In the first step, kraft lignin underwent a Friedel–Crafts alkylation reaction with 1,2‐dichloroethane in the presence of anhydrous aluminum chloride, yielding chlorinated lignin. In the second step, chlorinated lignin reacted with excess ethylenediamine in dimethylformamide at 80 °C for 7 h to introduce amino groups, forming aminated lignin. It was found that the equilibrium data were better represented by the Freundlich isotherm model, with saturated adsorption capacities reaching a maximum of 56 mg g^−1^ at pH 7.0. Thermodynamic parameters indicated that the adsorption of TNT on aminated lignin is an endothermic and spontaneous process. The pH plays a key role in the TNT adsorption capacity of aminated lignin. They also investigated the reusability of aminated lignin, showing more than 95% recovery after eight desorption–adsorption cycles using ethanol as the eluent.

Eutrophication and agrochemical runoff have made phosphate and glyphosate major environmental concerns. Li et al.^[^ ^105a^ ^]^ developed regenerable magnetic AL/Fe_3_O_4_/La(OH)_3_ adsorbents by incorporating Fe_3_O_4_ and La(OH)_3_ into aminated lignin, which was synthesized by grafting polyethyleneimine onto purified kraft lignin via the Mannich reaction involving formaldehyde under alkaline conditions at 60 °C. The composite exhibited excellent performance in removing both phosphate and glyphosate (Figure 8ci). These adsorbents achieved an adsorption capacity of 60 mg g^−1^ for phosphate and 84 mg g^−1^ for glyphosate at initial concentrations of 150 and 250 mg L^−1^, respectively. The composite is regenerable, providing a practical solution for agricultural wastewater remediation.

With growing pressure to mitigate climate change, lignin‐based adsorbents have also been developed for CO_2_ capture. N‐lignin has been explored as an adsorbent for CO_2_ capture. Recently, Fan et al.^[^ ^105b^ ^]^ prepared a nitrogen‐dotted lignin‐based hypercrosslinked polymer (Figure 8cii). Following nitrogen functionalization, the polymer exhibited a 120% enhancement of CO_2_ uptake. To further improve its performance in CO_2_ capture, the polymer underwent pyrolysis and carbonization, resulting in a final product with a remarkable CO_2_ uptake of 98 mg g^−1^. This innovative approach presents a sustainable and effective alternative for CO_2_ capture.

In summary, nitrogen functionalization significantly enhances the adsorption capacity of lignin by introducing electron‐rich functional groups, improving surface charge density, and enabling specific binding mechanisms for various pollutants. This versatile strategy enables lignin to compete with conventional adsorbents in diverse environmental applications, including heavy metal removal, dye treatment, agrichemical capture, and CO_2_ mitigation, offering a sustainable path for pollution control (**Table** **1**).

**Table 1:** Using N‐lignin for adsorption.

	Adsorbent	Adsorbate	Adsorption capacity (mg/g)	Ref.
Physical blending	Magnetic lignin‐chitosan composite	Methylene orange	330	[106]
	Lignin/chitosan composite beads	*p*‐nitrophenol	593	[39b]
	Lignin/chitin film	Methyl violate	1001	[44]
	Lignin/gelatin beads	Pb(II) ions	155	[107]
Grafting copolymerization	Lignin/PEI‐based nanoparticle	Ciprofloxacin	823	[108]
	Lignin/chitin crosslinked aerogel	Congo red	2960	[109]
	Lignin/chitosan/PVA crosslinked sponge	Hg(II) ions	664	[110]
	Lignin/PEI composite	Cr(VI) ions	898	[111]
Chemical modification	Chitosan‐amidated lignin aerogel beads	phosphate	131	[112]
	Aminated lignin/MIL‐101‐Fe‐NH_2_	Congo red	1495	[113]
	Iminated lignin	Pb^2+^ ions	116	[58]
	Carbonized quaternized lignin	CO_2_	98	[105b]
	Aminated lignin	2,4,6‐trinitrotoluen	56	[91]

### 3.4 Energy and Sensing Applications

Recent research has increasingly focused on N‐lignin as a sustainable and multifunctional material for advanced energy storage, energy harvesting, and sensing applications. Benefiting from its abundant redox‐active sites, electron‐donating nitrogen groups (e.g., amines, imidazoles, and quaternary ammonium), and renewable aromatic backbone, N‐lignin offers remarkable advantages in enhancing electrical conductivity, surface polarity, and interfacial compatibility with functional matrices. This functionalization significantly broadens the application potential of lignin in energy storage and sensor technologies.

**Figure 9:**
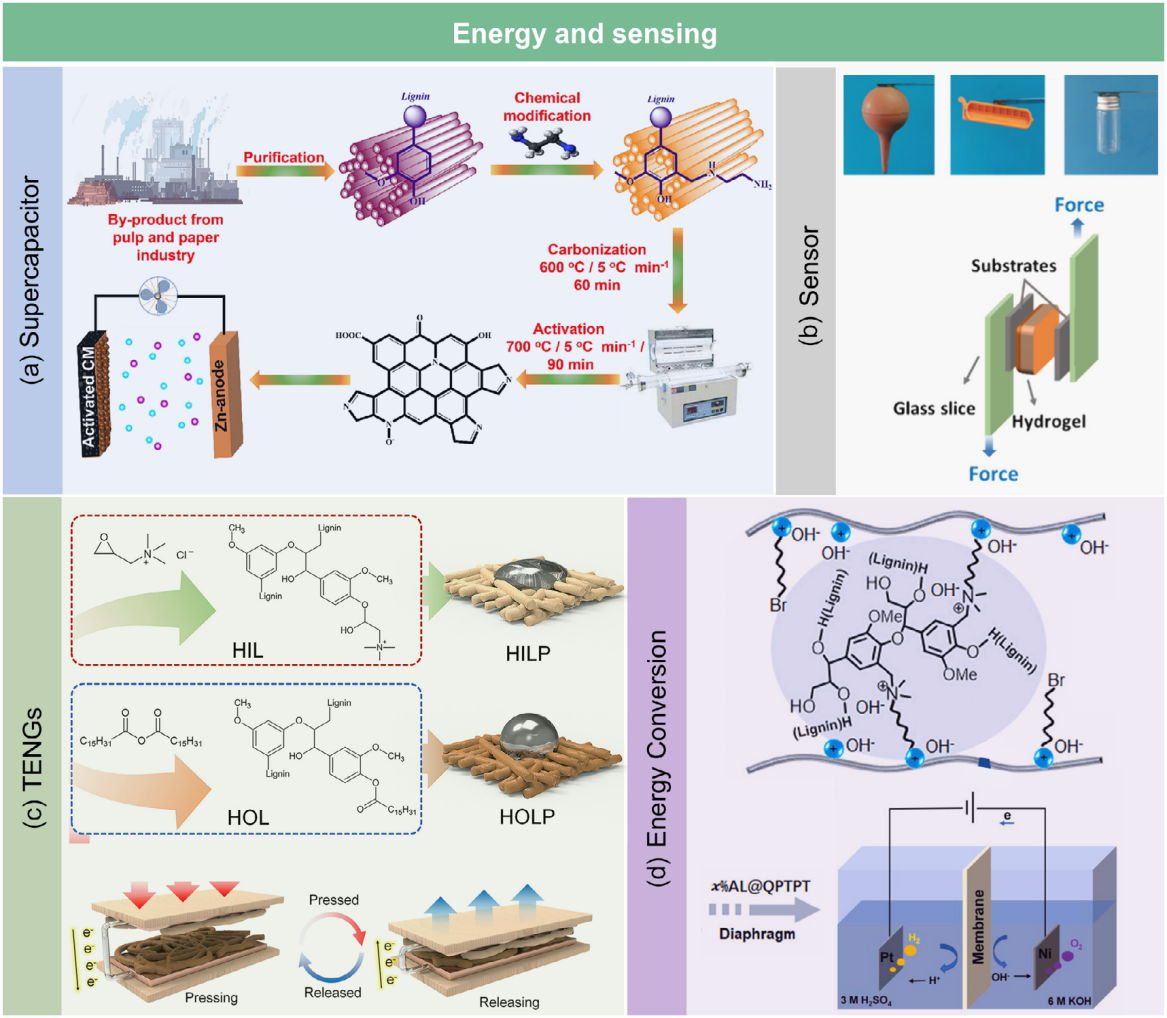
Energy and sensing applications of N‐lignin. a) Supercapacitors. Adapted with permission.^[^ ^92^ ^]^ Copyright 2023, Elsevier. b) Sensors. Adapted with permission.^[^ ^93^ ^]^ Copyright 2025, American Chemical Society. c) Triboelectric nanogenerators. Adapted with permission.^[^ ^95^ ^]^ Copyright 2023, Wiley‐VCH under CC BY 4.0 (https://creativecommons.org/licenses/by/4.0/). d) Energy conversation. Adapted with permission.^[^ ^96^ ^]^ Copyright 2025, Elsevier.

*Supercapacitors*. Recent studies have demonstrated the efficacy of N‐lignin‐derived materials as electrode components in high‐performance supercapacitors. For instance, Guo et al.^[^ ^92^ ^]^ developed pyrrolic‐N dominated carbon materials from aminated lignin through a Mannich reaction followed by carbonization and KOH activation (**Figure** **9**a). The resulting material exhibited a hierarchical porous structure with an ultrahigh surface area (2926.4 m^2 ^g^−1^) and high nitrogen content, significantly enhancing Zn‐ion storage capacity and charge‐transfer kinetics. As a cathode for Zn‐ion supercapacitors, it achieved an excellent specific capacity of 161.2 mAh g^−1^ and retained 94% capacity after 10 000 cycles, showcasing superior energy and power densities.

In a complementary study, Dong et al.^[^ ^16^ ^]^ fabricated a flexible all‐solid‐state supercapacitor using lignin‐containing cellulose nanofibrils reinforced with a lignin/polypyrrole (LS/PPy) interpenetrating network. The synergistic interaction between lignin's quinone/hydroquinone redox couple and conductive PPy resulted in outstanding areal capacitance (2567 mF cm^−2^), high coulombic efficiency (≈98%), and mechanical robustness. Notably, optimized lignin content (11.4%) was key to balancing conductivity, electrochemical activity, and structural integrity.

In summary, these studies highlight that nitrogen‐rich lignin derivatives enhance pseudocapacitive behavior, conductivity, and ion‐accessibility, offering sustainable alternatives to conventional carbon materials in next‐generation energy storage devices.

*Sensor*. N‐lignin has been successfully applied in both electrochemical and wearable sensing platforms, benefiting from its redox activity, surface tunability, and biocompatibility. One notable example is that Huang et al.^[^ ^93^ ^]^ developed a highly adhesive hydrogel sensor composed of aminated lignosulfonate and aminated cellulose nanocrystals through dynamic Schiff base bonding and hydrogen bonding interactions. The resulting hydrogel demonstrated high stretchability (>200%), short response time (50 ms), high gauge factor (up to 2.86), strong tissue adhesion (>20 kPa), and enhanced electron mobility (Figure 9b). It was capable of accurately monitoring joint movement, vocal cord vibration, and pulse pressure, and even used to power a self‐powered sensor circuit. This design showcases the potential of N‐lignin as a mechanically and electrically functional material for wearable health monitoring and self‐powered electronics.

In another approach, Gigli et al.^[^ ^94^ ^]^ constructed lignin‐chitosan hybrid nanoparticles by coating LNPs with protonated chitosan shells, forming a redox‐active core‐shell structure for electrochemical biosensing. The nitrogen‐rich lignin core, containing phenolic and aminated functionalities, acted as the primary redox mediator, while the chitosan shell modulated surface charge and hydrophilicity. By tuning the chitosan content, the researchers could precisely control the nanoparticle zeta potential, electrochemical responsiveness, and reactive oxygen species detection sensitivity (e.g., H_2_O_2_). The system demonstrated stable voltammetric redox peaks, indicating efficient electron transfer between the lignin core and target analytes. These features suggest strong applicability in point‐of‐care diagnostic platforms, especially for detecting oxidative stress or redox biomarkers in biological fluids.

In summary, these studies underscore the role of N‐lignin as a multifunctional interface material, capable of providing mechanical flexibility, electrical conductivity, and biochemical selectivity, which makes it highly suitable for the development of next‐generation wearable, implantable, or portable biosensors.

*Triboelectric nanogenerators (TENGs)*. N‐lignin has also been employed as a key component in biodegradable TENGs, where its electron‐donating groups (e.g., amines, hydroxyls, and sulfonic acids) enhance surface charge transfer and modulate triboelectric polarity. These systems highlight the dual role of N‐lignin as both an electrically active and mechanically supportive material for energy harvesting and self‐powered sensing. In a study, Jo et al.^[^ ^95^ ^]^ fabricated lignin/polycaprolactone nanofiber‐based TENGs via electrospinning, where hydrophilic nitrogen‐modified lignin (HIL) demonstrated enhanced tribopositive performance. By introducing quaternary ammonium groups into lignin, they significantly increased the surface energy and electrostatic potential, enabling efficient electron exchange with Teflon as the tribonegative pair (Figure 9c). The resulting HIL‐based TENG achieved a peak output voltage of over 95 V under a relatively low tapping force (9 N) and frequency (9 Hz), outperforming conventional biomass‐based TENGs in both efficiency and power density. Notably, the device maintained excellent cyclic stability over 100 000 tapping cycles, retained high mechanical strength due to improved fiber interactions, and powered 34 LEDs, highlighting its feasibility for wearable energy‐harvesting and self‐powered electronics. This study underscores the feasibility of utilizing N‐lignin as a high‐performance, eco‐friendly triboelectric material, offering a sustainable path toward self‐powered wearable electronics and green energy harvesting technologies.

*Energy conversion*. N‐lignin has also shown promising potential in electrochemical energy conversion, particularly in membrane‐based water electrolysis systems. Gu et al.^[^ ^96^ ^]^ developed aminated lignin via the Mannich reaction and utilized it as a crosslinker and radical scavenger in quaternized poly(terphenyl piperidinium)‐based membranes for amphoteric water electrolysis (Figure 9d). The resulting cross‐linked membranes exhibited excellent physicochemical properties, including high hydroxide (112 mS cm^−1^) and proton (210 mS cm^−1^) conductivities at 80 °C. Notably, when added 2% aminated lignin into the quaternized membrane, it demonstrated superior long‐term alkaline stability, maintaining 80% conductivity after 1500 h of immersion in 2 M KOH at 80 °C, along with robust mechanical properties (tensile strength of 34.4 MPa). When applied in a full electrolyzer configuration, this membrane enabled a current density of 954 mA cm^−2^ at 2 V and 80 °C and operated stably at 100 mA cm^−2^ for over 83 h with an initial energy consumption of 2.8 kWh Nm^−3^ for hydrogen production. These findings underscore the multifunctional role of N‐lignin, which not only enhances membrane durability and conductivity via radical scavenging and crosslinking but also facilitates sustainable and efficient hydrogen production in next‐generation electrochemical devices.

### 3.5 Other Emerging Applications

In addition to the functional applications discussed earlier, N‐lignin, with its improved physicochemical properties, such as increased solubility, enhanced surface activity, and improved reactivity toward various substrates, has found applications in several other fields. These include antifouling coatings, nitrogen‐based fertilizers, emulsifiers, and catalysis.

*Fertilizer*. Due to the inherently low nitrogen content of lignin, its direct use as a fertilizer poses significant challenges.^[^ ^97^ ^]^ However, nitrogen functionalization transforms lignin into a slow‐release, nutrient‐rich platform. For example, Jiao et al.^[^ ^98^ ^]^ successfully synthesized a highly efficient nitrogen fertilizer directly from lignin through amination. The resulting aminated lignin achieved a high nitrogen content (10%) and a low C/N ratio (6). Additionally, the N‐lignin exhibited gradual degradation and slow‐release properties, starting after 28 days. This study highlights the promising applications of lignin‐based fertilizers.

**Figure 10:**
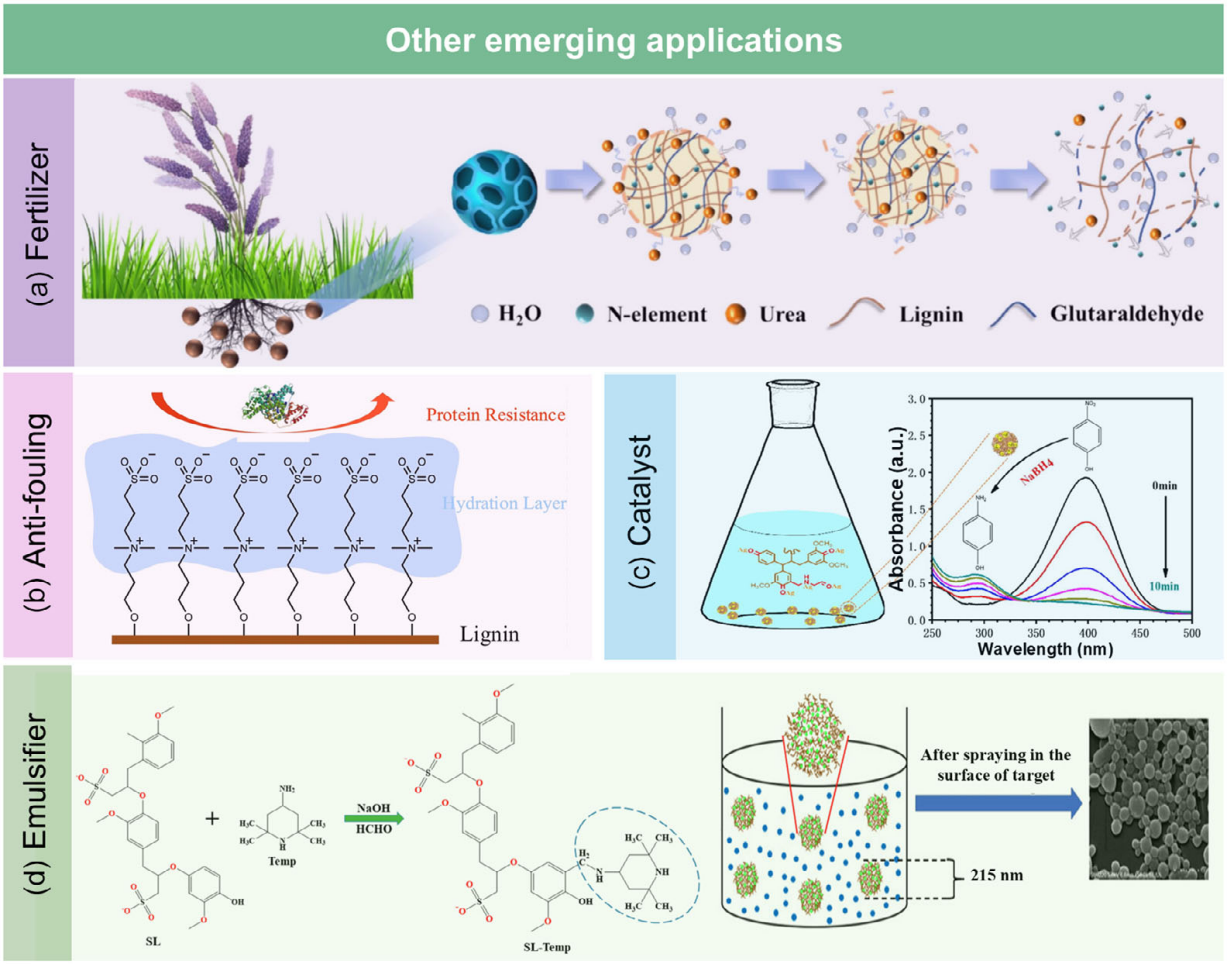
Emerging applications of N‐lignin in a) fertilizer. Adapted with permission.^[^ ^99^ ^]^ Copyright 2024, Elsevier. b) Antifouling. Adapted with permission.^[^ ^100^ ^]^ Copyright 2024, Elsevier. c) Catalyst. Adapted with permission.^[^ ^103^ ^]^ Copyright 2020, Elsevier. d) Emulsifier. Adapted with permission.^[^ ^104^ ^]^ Copyright 2019, American Chemical Society.

In a more recent work, Ji et al.^[^ ^99^ ^]^ developed a bifunctional slow‐release fertilizer by a simple one‐pot method (**Figure** **10**a). Softwood lignin was first dispersed in water and reacted with glutaraldehyde (40 wt%) at 85 °C for 2 h to introduce crosslinkable aldehyde groups. Subsequently, aqueous urea solution (50 wt%) was added and the mixture was stirred at 95 °C for another 2 h under alkaline conditions (pH 9.0), yielding porous microsphere‐shaped lignin‐urea composites (L‐UX) with tunable morphology and nitrogen content. This fertilizer achieved an impressive nitrogen loading (66.2%) and exhibited prolonged nutrient release for up to 288 h. Moreover, L‐UX significantly improved the water retention capacity of saline‐alkaline soils and promoted wheat germination under arid and saline conditions. A comprehensive life cycle assessment revealed that L‐UX had a lower environmental impact compared to conventional urea‐based fertilizers, underscoring its potential as a green and sustainable fertilizer in harsh agricultural environments.

*Antifouling.* The introduction of nitrogen‐containing groups imparts hydrophilicity and zwitterionic character to lignin, enabling it to function as an effective antifouling agent. Conventional antifouling agents are often costly and challenging to synthesize, whereas modified lignin resents a viable alternative for such applications. For example, Zhou et al.^[^ ^100^ ^]^ fabricated an antifouling nanofiltration membrane using aminated lignin. In their study, lignin was chemically modified and incorporated into the interfacial polymerization process to improve the hydrophilicity and antifouling properties of the membrane. The results demonstrated that the addition of modified lignin improved the membrane permeability, maintained high Na_2_SO_4_ rejection, and significantly enhanced antifouling performance. In another study, An et al.^[^ ^101^ ^]^ synthesized zwitterionic lignin, containing both cationic and anionic functional groups, and applied it to protein resistance. The functionalized lignin showed excellent protein resistance, with bovine serum albumin adsorption reduced to 27.0 ± 0.9 μg cm^−2^, a significant decrease compared to the 114.3 ± 8.1 μg cm^−2^ observed for unmodified lignin.

In another study, An et al.^[^ ^101^ ^]^ synthesized tailor‐made zwitterionic lignin through a two‐step grafting process (Figure 10b). First, kraft lignin was aminated using 3‐dimethylamino‐1‐propyl chloride hydrochloride in alkaline pyridine at 50 °C for 24 h to introduce tertiary amine groups. Then, the aminated lignin reacted with 1,3‐propanesultone in tetrahydrofuran at 70 °C for 24 h to graft sulfonic acid groups via a ring‐opening reaction. This process successfully introduced zwitterionic sulfobetaine structures into lignin, resulting in enhanced hydrophilicity and a strongly negative surface charge. The final zwitterionic lignin showed significantly reduced protein adsorption (27.0 ± 0.9 μg cm^−2^), attributed to the formation of a dense hydration layer and electrostatic repulsion.

*Catalyst.* Nitrogen functional groups also endow lignin with coordination and redox capabilities, enabling its use as a catalyst or catalyst support. For example, Chen et al.^[^ ^102^ ^]^ developed lignin‐based catalysts for the removal of organic pollutants, such as methylene blue and 4‐nitrophenol. Specifically, a lignin Schiff base with enhanced chelation ability toward copper ions was synthesized. The resulting Schiff base‐Cu complex served as a catalyst for the reduction of methylene blue and 4‐nitrophenol. This approach presents a simple and effective method for using lignin as a high‐efficiency catalyst.

In another study, Pang et al.^[^ ^103^ ^]^ prepared a lignin‐based composite catalyst by in situ reducing silver ions using AAL as both a reductant and stabilizer (Figure 10c). The AAL was synthesized via Mannich reaction of alkaline lignin, ethylenediamine, and formaldehyde (1:1:9 molar ratio) at 70 °C for 4 h under alkaline conditions (5 wt% NaOH). Subsequently, Ag^+^ (200 mg L^−1^) was added and reduced at 80 °C for 3 at pH 6–7, yielding monodispersed AgNPs (≈17.1 nm) embedded in the lignin matrix. The resulting AgNPs/AAL catalyst exhibited excellent catalytic activity toward the reduction of 4‐nitrophenol to 4‐aminophenol (k = 0.231 min^−1^), and maintained >98% efficiency after eight reuse cycles, demonstrating high stability and recyclability.

*Emulsifier.* N‐lignin has also demonstrated excellent emulsifying properties. Zhou and colleagues^[^ ^104^ ^]^ synthesized amine‐grafted lignin and used it as an emulsifier to prepare a green emulsifiable concentrate of avermectin, which exhibited good emulsifying performance and storage stability. Moreover, the avermectin emulsifiable concentrate showed an impressive release equilibrium time, which was 5.3 times longer than that of the commercial emulsifiable concentrate (Figure 10d).

These emerging applications illustrate the versatility of N‐lignin across multiple domains, particularly where sustainability, multifunctionality, and processability are valued. Notably, the structural tunability of lignin enables tailored physicochemical properties that directly correlate with end‐use performance, such as hydrophilicity, redox activity, and interfacial behavior. Nitrogen functionalization does not merely improve lignin's reactivity but serves as a strategic platform to bridge biomass‐derived materials with advanced functional systems, especially in fields traditionally dominated by petroleum‐based polymers or inorganic catalysts.

## 4 Future Perspectives

N‐lignin represents a promising advancement in the field of lignin valorization, offering enhanced properties that expand its applicability across diverse industrial sectors. Despite notable progress, several key challenges must be overcome to enable its large‐scale implementation. Future research efforts should prioritize improving functionalization efficiency, addressing structural heterogeneity, developing high‐performance applications, and optimizing scalability and sustainability of the processes.

First, significant attention should be directed to enhancing nitrogen‐functionalization strategies. While existing methods, such as chemical grafting and physical blending, have demonstrated efficacy, challenges remain in achieving efficient nitrogen incorporation. The development of innovative catalytic and bio‐based functionalization methods, such as enzymatic transformations and green chemistry approaches, could offer more sustainable and selective modifications while minimizing environmental impact. Additionally, exploring synergistic modifications, such as dual functionalization with nitrogen and other elements (e.g., sulfur or phosphorus), could further improve the reactivity and performance of the material in targeted applications.

Second, tackling intrinsic structural heterogeneity of lignin is crucial for improving process consistency and material performance. Variations in molecular weight, functional group distribution, and polymeric structure pose significant challenges in achieving uniform N‐lignin materials. Fractionation techniques, such as selective precipitation, membrane filtration, and chromatographic separation, could enable the isolation of well‐defined lignin fractions with enhanced reactivity. Additionally, computational modeling and machine learning approaches hold promise for predicting modification pathways and optimizing functionalization conditions.

Third, further efforts should focus on developing high‐performance materials tailored for specific applications to fully leverage the potential of N‐lignin. While its applications in antioxidants, antibacterial agents, adsorbents, fertilizers, and emulsifiers have been extensively studied, additional advancements are needed to optimize mechanical strength, thermal stability, and compatibility with industrial matrices. For instance, nanoengineering approaches, such as the development of lignin‐based nanocomposites, hybrid materials, and lignin‐derived nanogels, could significantly enhance the functional properties and broaden the scope of applications.

Fourth, scalability and process economics remain critical barriers to commercialization. Although laboratory‐scale studies have demonstrated promising results, translating these findings to industrial‐scale production requires focusing on cost‐effective processing, feedstock availability, and energy‐efficient methodologies. Exploring advanced reactor design, continuous‐flow processing, and solvent recovery systems could enhance yield and improve sustainability. Additionally, conducting techno‐economic assessments and life cycle analyzes is essential for evaluating the feasibility of N‐lignin in comparison to existing commercial alternatives.

Finally, regulatory compliance and commercialization strategies should be considered in future research. The incorporation of N‐lignin into applications such as biomedical products, food packaging, and agricultural fertilizers requires thorough toxicological assessments, biocompatibility studies, and environmental impact analyzes. Establishing international standards and securing regulatory approvals will be crucial for achieving market acceptance and facilitating widespread adoption.

## 5 Conclusions

N‐lignin has emerged as a versatile and sustainable platform for applications in biomedicine, agriculture, environmental remediation, energy, and electronics. The introduction of nitrogen‐containing functional groups has significantly improved chemical reactivity, solubility, and interaction with various substrates, thereby enhancing the performance of material across multiple domains. This review provides a comprehensive overview of current functionalization techniques, recent advances in applications, and the key challenges that must be addressed to enable large‐scale utilization.

From an application perspective, N‐lignin has demonstrated remarkable potential in environmental remediation, sustainable agriculture, energy harvesting and sensing, as well as biomedical engineering. To fully realize its potential in commercial applications, future studies should focus on optimizing material performance, biocompatibility, and degradation kinetics. Moreover, the integration of lignin‐based functional materials into polymer composites, coatings, and next‐generation biomaterials offers exciting opportunities for future advancements.

Overall, N‐lignin represents an emerging class of bio‐based materials with the potential to drive sustainable innovation across multiple industries. Moving forward, interdisciplinary collaboration between chemists, material scientists, engineers, and industrial stakeholders will be critical to overcoming existing challenges and unlocking the full potential of this renewable resource. With ongoing advancements in processing technologies, functionalization techniques, and application development, N‐lignin is poised to become a key component in the transition toward sustainable and high‐performance bio‐based materials.

## Conflict of Interest

The authors declare no conflict of interest.
